# Report on a large animal study with Göttingen Minipigs where regenerates and controls for articular cartilage were created in a large number. Focus on the conditions of the operated stifle joints and suggestions for standardized procedures

**DOI:** 10.1371/journal.pone.0224996

**Published:** 2019-12-26

**Authors:** Markus L. Schwarz, Gregor Reisig, Andy Schütte, Kristianna Becker, Susanne Serba, Elmar Forsch, Steffen Thier, Stefan Fickert, Tamara Lenz, Christel Weiß, Svetlana Hetjens, Frederic Bludau, Friederike Bothe, Wiltrud Richter, Barbara Schneider-Wald

**Affiliations:** 1 Section for experimental Orthopaedics and Trauma Surgery, Orthopaedic and Trauma Surgery Centre (OUZ), Medical Faculty Mannheim, Heidelberg University, Mannheim, Germany; 2 Interfaculty Biomedical Facility, Heidelberg University, Heidelberg, Germany; 3 Department of Experimental Pain Research, Medical Faculty Mannheim, Heidelberg University, Mannheim, Germany; 4 Sportchirurgie Heidelberg, Klonz—Thier–Stock, ATOS Klinik Heidelberg, Heidelberg, Germany; 5 Sporthopaedicum Regensburg/Straubing, Straubing, Germany; 6 Statistical Consulting, Mannheim, Germany; 7 Department of Medical Statistics, Medical Faculty Mannheim, Heidelberg University, Mannheim, Germany; 8 Research Centre for Experimental Orthopaedics, Heidelberg University Hospital, Heidelberg, Germany; Drexel University, UNITED STATES

## Abstract

The characterization of regenerated articular cartilage (AC) can be based on various methods, as there is an unambiguous accepted criterion neither for the natural cartilage tissue nor for regenerates. Biomechanical aspects should be considered as well, leading to the need for more equivalent samples. The aim of the study was to describe a large animal model where 8 specimens of regenerated AC can be created in one animal plus the impact of two surgeries on the welfare of the animals. The usefulness of the inclusion of a group of untreated animals (NAT) was to analyzed. Based on the histological results the conditions of the regenerates were to be described and the impact on knee joints were to be explored in terms of degenerative changes of the cartilage. The usefulness of the statistical term “effect size” (ES) will be explained with histological results. We analyzed an animal model where 8 AC regenerates were obtained from one Göttingen Minipig, on both sides of the trochleae. 60 animals were divided into 6 groups of 10 each, where the partial thickness defects in the trochlea were filled with matrices made of Collagen I with or without autologous chondrocytes or left empty over the healing periods of 24 and 48 weeks. One additional control group consisting of 10 untreated animals was used to provide untouched “external” cartilage. We harvested 560 samples of regenerated tissue and “external” controls, besides that, twice the number of further samples from other parts of the joints referred to as “internal” controls were also harvested. The animals recovered faster after the 1^st^ operation when the defects were set compared to the 2^nd^ operation when the defects were treated. 9% of all animals were lost. Other complications were for example superficial infections, seroma, diarrhea, febrile state and an injury of a claw. The histological results of the treatments proved the robustness of the study design where we included an “external” control group (NAT) in which the animals were not operated. Comparable significant differences between treated groups and the NAT group were detected both after ½ year and after 1 year. Spontaneous regenerated AC as control revealed differences after an observation time of nearly 1 year. The impact of the treatment on cartilage adjacent to the defect as well as the remaining knee joint was low. The ES was helpful for planning the study as it is shown that the power of a statistical comparison seems to be more influenced by the ES than by the sample size. The ranking of the ES was done exemplarily, listing the results according to their magnitude, thus making the results comparable. We were able to follow the 3 R requirements also in terms of a numerical reduction of animals due to the introduction of a group of untreated animals. This makes the model cost effective. The presented study may contribute as an improvement of the standardization of large animal models for research and regulatory requirements for regenerative therapies of AC.

## Introduction

Treatment of articular cartilage defects is provided using regenerative therapeutic methods with [1] and without cells [2–4]. However, the search for the ideal treatment of an articular cartilage lesion is not finished yet [5–8].

In a recent survey, we found that the sheep is the most used animal in the field of regenerative cartilage research, followed by the swine and the goat [9]. However, large animal models are cost-intensive in terms of the acquisition, surgery and housing. This is true in particular for swine, which require individual housing [10], and appropriate facilities are limited.

Schneider-Wald et al. [9] also pointed out, that several different strategies of analyses of the regenerates exist and it seems that their number is growing. Most of the methods consume sample material. Specimens processed for histological analysis, for example, cannot be used for biomechanical tests or *vice versa* as both will destroy the specimen and are relied on a pristine tissue structure. To our knowledge, there is no single test to sufficiently characterize the properties of articular cartilage and regenerates and the number of useful test procedures rather seems to be growing. Meanwhile, biological issues and biomechanical aspects of the analyses become more important [9, 11, 12]. Kim et al. [13] recommend that a “plethora of outcomes” should be assessed to fully analyze the treatment of articular cartilage.

On the one hand, large but expensive animal models seem to be mandatory, but on the other hand a lot of equal samples are required to characterize the regenerated articular cartilage. For that reason we modified and enhanced a large animal model as, for example, described by [14], which focuses on the trochlear groove of the stifle joint of Göttingen Minipigs (GM). In our opinion, this model has the potential to create regenerates in a large number and in a reproducible and reliable quality [15, 16]. In this context we developed instruments for reproducible defect setting in cartilage and for harvesting osteochondral specimens [17].

Whatever type of animal or assessment is applied, the quality of the results depends particularly on the quality of controls, because cartilage lying adjacent to defects created for regenerative treatment can be altered [18, 19]. Thus, controls gathered from the same joint might not be appropriate to determine the real effect of the treatment. A different possibility is the use of the opposite joint [18] but in this case, an asymmetrical load bearing of the limbs has to be considered as that could lead to damages at one site. Another solution could be the inclusion of non—operated animals [20] who would serve as an “external” control (“NAT group”). The welfare of an untreated group can be guaranteed if appropriate housing is provided. This procedure is in line with the three “Rs” in the ARRIVE guidelines [21] as it helps to reduce the number of treated animals but at the same time avoids the reduction of the validity of the results [22–24].

Another important question is how to manage and prepare statistics for a variety of results from different analyses which are then used as parameters to determine which procedure reflects the assessed issue appropriately.

In a study where more than one parameter is of primary importance, possibly with measurements performed under different conditions and at different times, the challenge in the planning phase is to determine an acceptable sample size. The procedure for calculating an acceptable sample size that guarantees sufficient power for the study is much the same as in any other study with only one parameter. Nevertheless, it is desirable to attain power for a maximum number of parameters of importance in the study. By considering effect size (ES) [25] in general, it is possible to arrive at an overall sample size for the total experiment that would also satisfy power considerations in a satisfactory manner. The ES should also help to identify the suitability of any analysis compared with others, regarding a classification in “small”, “medium” and “large” [26].

The aim of this publication is to describe a large animal model where 8 samples of regenerated articular cartilage can be created in one animal, furthermore to report on the welfare of the animals and the complications occurred. The outcomes of the treatments are described histologically. We communicate effects on the articular cartilage close to the defects and the knee joints analyzed by radiographs, macroscopically and histologically. In addition, we suggest a statistical procedure for calculation of the sample size and for conclusive evaluation.

With the presentation of the study and our experiences we intend to contribute to standardization of the research for regenerative therapies of articular cartilage and to regulatory requirements in conclusion.

## Material and methods

### Legal regulations, animals and housing

The study was approved by the ethical committee of the Regierungspräsidium

Karlsruhe (Abteilung 3—Landwirtschaft, Ländlicher Raum, Veterinär- und

Lebensmittelwesen, Karlsruhe, Germany) with the number: AZ 35–9185.81/G-6/11.

The declaration of killing of one Goettingen Minipig is recorded under the number T-56/10.

79 skeletally mature female Goettingen Minipigs (Ellegaard, Dalmose, Denmark and Versuchsgut Relliehausen, Univ. of Goettingen, Germany) were included in the study, 2 served as pilot animals. The animals included in the study had a mean age of 38 month (±7 month; median 38 month, range: 24–50 month). The mean weight was 53.7 kg ± 9.5 kg (median 54 kg, range: 33–74 kg) at the beginning of the trial.

The experiments were performed in the Interfaculty Biomedical Research Facility (IBF, Ruprecht-Karls-Universität Heidelberg, Heidelberg, Germany) under supervision of the local animal welfare officer. Throughout the study, visitations were recorded in terms of pain or other signs of discomfort and gait patterns to detect any events.

The animals were housed in boxes alone or together when possible. Sight contact, noise and smell exchange was possible between the animals. Animals were separated after surgery and in the case of quarrel. Water was available *ad libidum* and they were fed once a day (Minipig Standard Diet, SDS Deutschland, Ludwigshafen, Germany).

After the 2nd operation and removal of the sutures the animals were taken to an external facility until the end of the study.

### Study design

The study was based on two pilot animals, 6 experimental groups and one control group (NAT) (Table 1). The observations times were 2 weeks for the pilot and 24 and 48 weeks for the treated animals and 24 weeks for the NAT group (Table 1). The NAT group consisted of untreated animals and their pristine articular cartilage served as so-called “external” control [20]. All groups consisted of 10 pigs, following a sample size calculation (Table 1).

**Table 1:** The groups in the presented study. Two healing periods for the treated animals were assessed, one of approximately half a year (24 weeks) and the other of approximately one year (48 weeks). The NAT group consisted of animals that received no surgical treatment; their articular cartilage served as an “external control”. The groups with animals in which matrices made out of Col 1 were implanted without cells (M24w and M48w) can be interpreted as verum or control groups for the cell loaded implants (MC24w and MC48w). The groups with empty defects (E24w and E48w) can be seen as control for the groups with implants as well as control for the animal model in terms of the critical size of the created defects.

short name	MC24w	M24w	E24w	MC48w	M48w	E48w	NAT
type of implant	matrix with autologous chondrocytes	matrix without cells	defects left empty	matrix with autologous chondrocytes	matrix without cells	defects left empty	none
observation period	24 weeks	24 weeks	24 weeks	48 weeks	48 weeks	48 weeks	24 weeks
number of animals	n = 10	n = 10	n = 10	n = 10	n = 10	n = 10	n = 10

**Fig 1:**
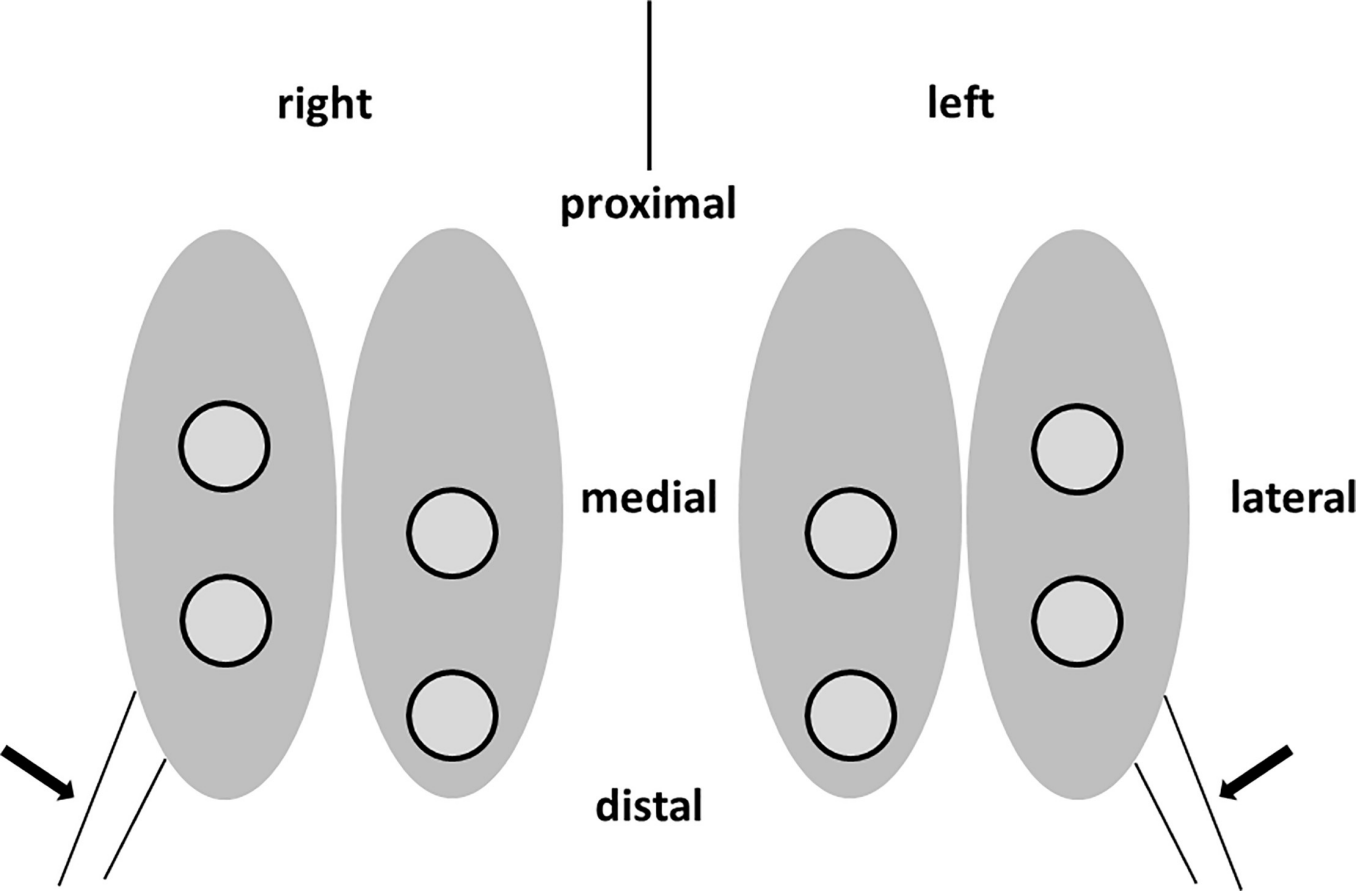
Schematic depiction of the allocation of the defects (circles) on both trochleae of one animal. The arrows indicate the tendon of the m. extensor digitorum longus [28], which can be helpful as anatomic landmark. The lateral distal defects were set first in line with the virtual extension of the tendon. Then the other defects were placed in an offset pattern.

**Fig 2:**
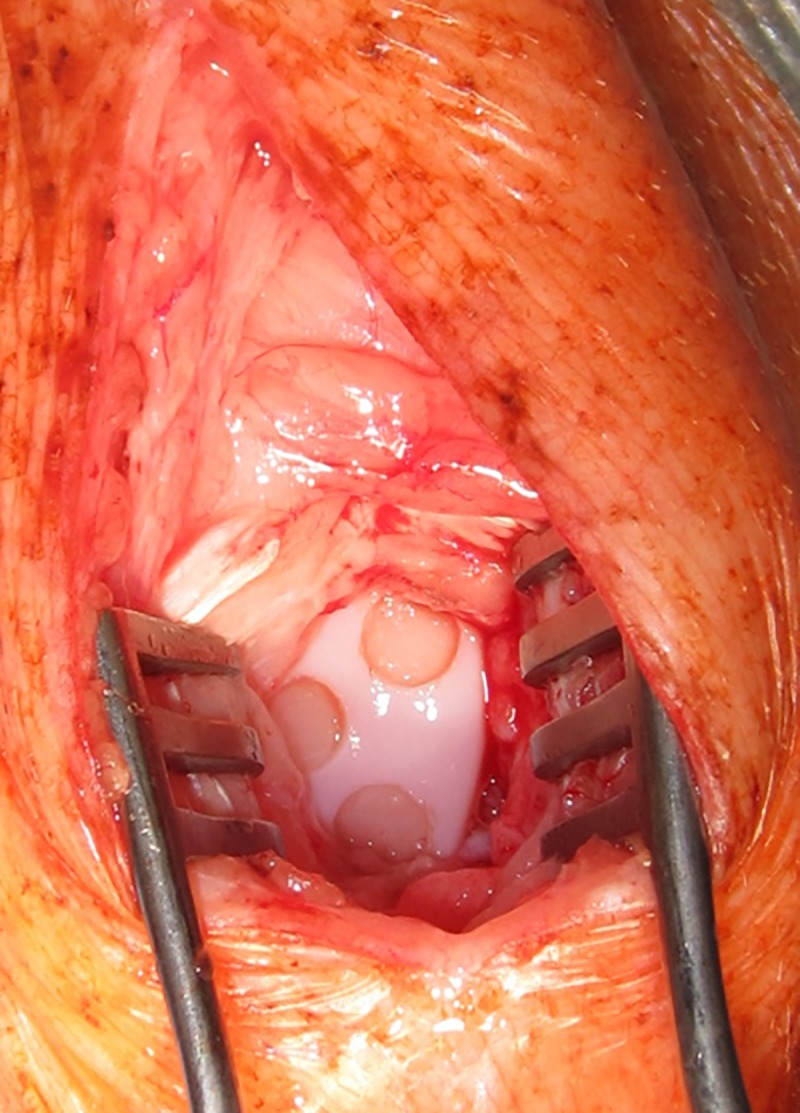
View into a left knee joint after setting the defects. The retractor is orientated to the foot (lower edge of the image). The defects number one, two and three are exposed. The fourth, distally located at the medial facet is covered by soft tissue (see Fig 1).

Aware that the critical size for a defect was a minimum of 5 mm in diameter [14] cited by [27], we set 4 defects, 6 mm in diameter, bilaterally on both facets of the trochlea, as shown in Figs 1 and 2.

Untreated areas of the trochlea and the other parts of joints are referred to as “inner” controls if required. In all groups, areas where controls were harvested and defect locations were identical.

### Preparation for surgery, anesthesia and killing

Over approximately 2 weeks of acclimatization the animals received feed daily until the day before surgery. Water was always available *ad libitum*.

The animals were premedicated with an intramuscular injection of 10mg/kg ketamine (Ketanest S 25mg/ml Injekt.fl. 10ml; Pfizer, Berlin, Germany), 4 mg/kg azaperone (Stresnil®, Lilly Deutschland GmbH, Bad Homburg, Germany) and 1mg/kg midazolam (hameln pharma plus gmbh, Hameln, Germany). Blood samples were taken from the ear vein before venous indwellings (BD Venflon^™^ Pro Safety Catheter 22G, Becton Dickinson, Helsingborg, Sweden) were placed at both ears (one each). After intravenous administration of 1mg/kg propofol (Propofol 2%, Fresenius Kabi Deutschland GmbH, Bad Homburg, Germany), the animals were intubated and anaesthesia was performed with 0.8–1.2% inhalational isoflurane (Forene®, Abbott GmbH, Wiesbaden, Germany) under artificial respiration.

Before the pigs were transferred into the operating theatre we washed the hind limbs carefully with soap and water and the bristles in the knee joint region were removed with an electric razor (Aesculap Suhl GmbH, Suhl, GermanyTyp). Then we covered the feet of the swine with tissue towels (Vileda Lochtuch, Vileda GmbH, Weinheim, Germany) which were fixated strongly with plaster (Leukoplast®, BSN medical GmbH, Hamburg, Germany).

For perioperative infection prophylaxis, an infusion of 2 mg cefazolin (Basocef, Deltaselect, Germany) was administered [29].

The pain management was administered with fentanyl as required. Before the anesthesia was terminated 50 mg fentanyl (Fentanyl-Janssen, Janssen-Cilag GmbH, Neuss, Germany) and 4 mg/kg carprofen (Rimadyl ®, Pfizer GmbH, Berlin, Germany) were administered. Carprofen was applied postoperatively at signs of pain, or buprenorphin (Temgesic®, Indivior UK Ltd., Slough Berkshire, UK) if necessary.

At the scheduled date of killing the animals were inspected for their gait pattern before they were premedicated as described above. Blood samples were taken again. A venous indwelling was placed in an ear vein and the animals were killed by injection of min. 60 ml of saturated KCl i.v. under general anaesthesia with propofol.

EKG control (zero line), missing heartbeat, auscultatory and corneal reflexes determined death. After death, we carried out an autopsy to exclude diseases or alterations of the inner organs similar to [30]; brain and spinal cord were not assessed.

### Surgical procedure

We operated the animals in a supine position which was stabilized using a vacuum mattress. When the animals were connected to the artificial respirator (Narkomat, Heyer, Bad Ems, Germany), EKG (Lohmeier M111, München, Germany) and pulse oximeter (Lohmeier M211-371, München Germany), the legs were disinfected with Softasept N (B.Braun, Melsungen, Germany) three times each.

The sterile covering of the animal for surgery followed a standardised procedure. It was important to fix the “Stockinets” (Lohmann & Rauscher GmbH & Co. KG, Neuwied, Germany) covering the feet close to the ankle joint in a secure manner to avoid slippage during surgery, thus revealing unsterile areas. Sterile material for surgery was used to cover the body of the pig (Foliodrape® Extremitäten-Set III, Paul Hartmann AG, Heidenheim, Germany; Adhesive Tape, Medline International Germany GmbH, Kleve, Germany; Side Drape with Adhesive and Adhesive Tape, Cardinal Health, Dublin, USA).

The right leg was covered with a sterile towel, as we started with the surgery of the left leg and *vice versa* the left leg was covered, when the right leg was operated on.

### Defect setting, 1^st^ surgery

The joint was accessed by arthrotomy through the *ligamentum patellae* as described by [14] and [27].

Both in the 1^st^ and the 2^nd^ operation, the patellar tendon and the inferior patellar fat pad were split. Parts of the latter had to be resected in several cases under careful cauterization for haemostasis for a clearer view and accessibility during both operations.

**Fig 3:**
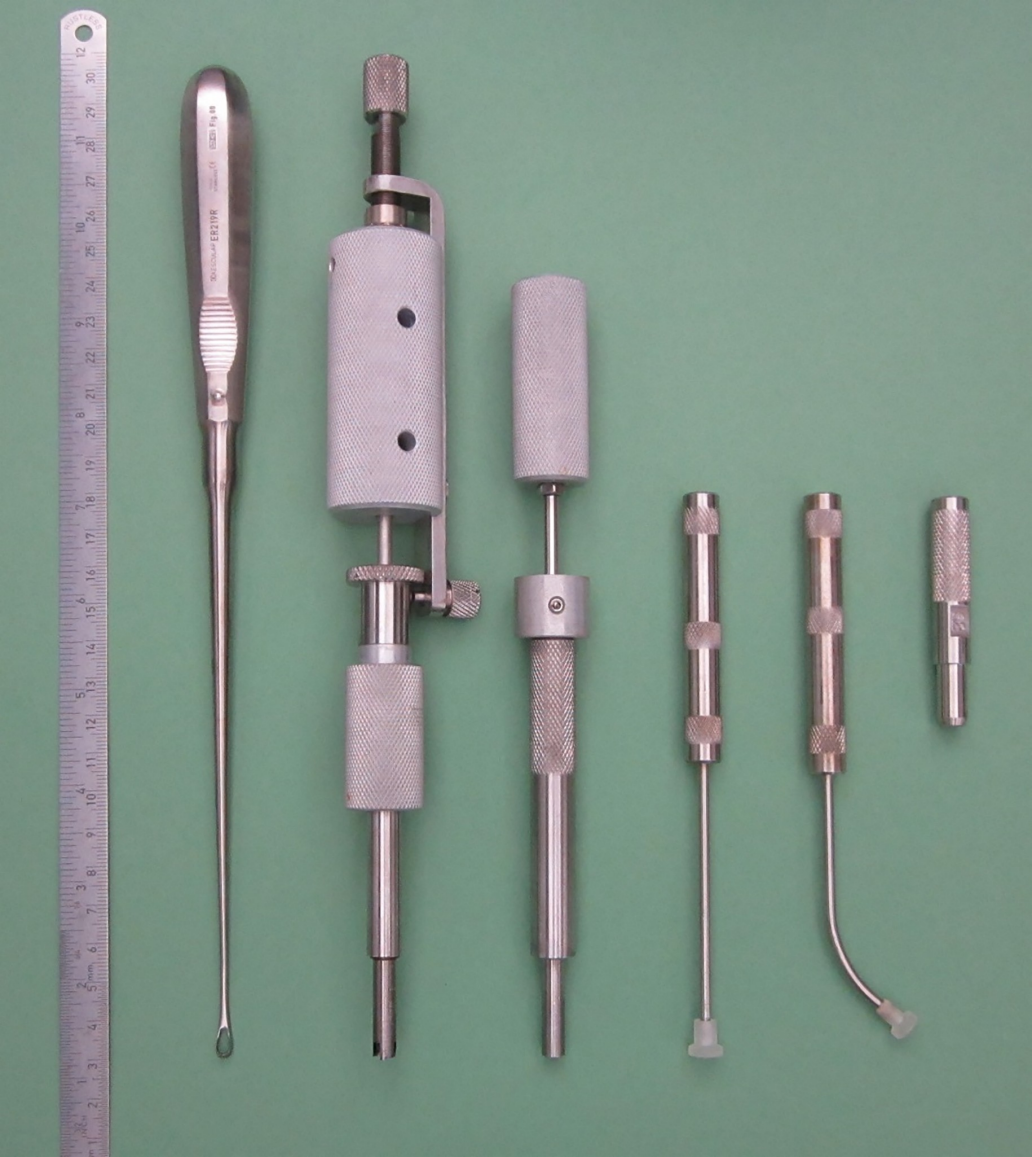
The instruments for setting from left to right: The curette, the crown mill and the front mill are displayed, which were described by Schwarz et al. [17] more in detail. The instruments for setting the scaffolds were equipped with a straight or curved shaft and two different shaped tappets, one concave and one flat on top (not visible). The instrument on the right is the punch used to create the implants; punches were provided with inner diameters varying from 6.2 mm to 6.7 mm.

The trochlea was exposed using a Weitlaner self-retaining retractor [31] and the accessible areas of the trochlea were identified by flexion and extension of the joint (Fig 1). The defects were set with special tools (Fig 3) in a reproducible manner. The depth was adjusted to 0.5 mm via ultrasound, according to prior pilot measurements, literature research and the situation *in situ*. Haptic feeling could reveal if cartilage or bone was cut by the so called crown mill as described by our working group [17]. The integrity of the subchondral lamella was respected, avoiding the appearance of blood points in general; residual tissue at the bottom of the defect was removed with a curette (Uterine curette ER219R, 4.5mm, Aesculap, Tuttlingen, Germany) sharpened on both sides [17]. Thus, an injury of the subchondral lamella could be prevented and “partial thickness defects” [32] were created as a result. After we had finished the surgery at the left side we continued with the right side, using a newly prepared set of instruments and clothing (see below).

After the defects had been set, the joint was rinsed with 0.9% NaCl (B.Braun, Melsungen, Germany). Then the joint was closed in layers using 2–0 Vicryl sutures (Ethicon, Norderstedt, Germany). For the cutaneous suture, Ethilon II (Ethicon, Norderstedt, Germany) was used. The surface of the wound was disinfected with Softasept N and afterwards sealed with aluminium spray (SanDitan®, Veyx Pharma GmbH, Schwarzenborn, Germany).

Smear tests were taken according to a hygiene plan.

For the fabrication of the collagen matrices seeded with autologous chondrocytes for the MC48w and MC24w groups, removed cartilage from the defect was stored in Dulbeccos phosphate buffered saline (DPBS, Life Technologies GmbH, Darmstadt, Germany) with 1% Gentamycin (A2712, Biochrom GmbH, Berlin, Germany) and shipped at 4°C to the company involved in this study (Fa. Amedrix GmbH, Esslingen, Germany).

### Implants

The implants were prepared and provided by the Fa. Amedrix, Esslingen, Germany, ready for implantation. The use of Col I as scaffold for regeneration of articular cartilage is described previously in several publications. The scaffolds are made out of Col I isolated from rat tails [33]. The scaffolds for the MC24w and the MC48w groups were laden with 2.5 x 10^4^ cells / ml.

### Defect filling, 2^nd^ surgery

An interval of 7 to 14 days between the 1^st^ and the 2^nd^ operation was necessary for recovery of the animals and the processing of the matrix by seeding them with the autologous chondrocytes in the MC24w and MC48w groups by the manufacturer (Fa. Amedrix). The same approach to the joint was used as in the 1^st^ operation. Regenerated tissue in the defect area was removed using the modified uterus curette and a chisel, 4 mm in width (Lambotte FL650R, Aesculap, Tuttlingen, Germany). Afterwards, the defects were filled according to the protocol (Table 1). The collagen matrix was prepared and shipped in Dulbeccos PBS at -20°C by the Fa. Amedrix for the M24w and M48w groups. Before implantation, they were thawed for processing. The chondrocyte laded collagen matrices, provided for the MC24w and MC48w groups were prepared for implantation and shipped in Dulbeccos PBS at +2°C—+8°C by the Fa. Amedrix in special containers.

We provided custom made hollow cutters with diameters ranging from 6.2 mm to 6.7 mm, allowing an adjustment of the size of the matrices to the size of the defects (Fig 3). The implants were adjusted to the estimated volume of the defect regarding the diameter of the defect, in particular, as in some cases the circularity of the defect was irregular, leading to an increase in size of the defect volume as a consequence. Thus a larger implant helped to cover the defect completely in some cases.

The matrices were then fixed, using Tissucol fibrin glue (Baxter, Unterschleißheim, Germany) and compressed from 2 mm to a height of ca. 0.5mm according to the instruction of the manufacturer. For this procedure we designed and built a custom made tappet with a concave surface (Fig 3) which allowed us to center the constructs into the defects preventing them of slipping out and compressing of the matrix. We then smoothed down the surfaces of the implants to the level of the surrounding cartilage surface using a tappet with a flat and polished surface (Fig 3). Protuberant matrix material was removed carefully.

The joint was bent and stretched at least 5 times after implantation to control the primary stability of the matrices. The further proceedings of wound closure were the same as described above.

### Hygiene plan

According to experiences in the pilot phase (see results), we followed a customized hygiene plan including smear tests in the 1^st^ and 2^nd^ surgery.

We prepared and sterilized a new set of surgical instruments for each operation site of each knee joint, furthermore the surgeons changed their mask and surgical cap, and disposable surgical gowns were used to assure sterility.

Furthermore, the non—operated leg was covered by a sterile cloth to prevent contamination.

The use of a self-adhesive incise foil (OPSITE Incise Drape, Smith & Nephew GmbH, Hamburg, Germany) covering the skin during the operation was to help reduce the contamination risk.

The wounds were covered with aluspray after closing in addition to dressing the wound with compresses and plaster. The aluspray covering was renewed, if necessary, later on.

### Harvesting of the samples and X-rays

The hind legs of each animal were disarticulated in the hip joint and the leg was cut above the ankle with a handsaw. The skin in the region of the operated knee joint was left intact.

The specimens were immediately transported to the laboratory in plastic bags labeled with the side and the ID number of the animal for further preparation. There, X-rays were taken in two planes in the ap and lateral view with the Faxitron (Faxitron cabinet x-ray system, Faxitron x-ray corporation, Buffalo grove, Illinois, US; and FUJIFILM IP Kassette CC, FUJIFILM Germany, Düsseldorf, Germany) or with a Siemens Axiom Aristos MX (Siemens Healthcare GmbH, Erlangen, Germany). The radiographs were stored in the PACS system and viewed with the syngo Imaging V35 System (Siemens AG, Medical Solutions Image and Knowledge Management, Erlangen, Germany).

Knee joints were opened according to the protocol. If sterile samples had to be isolated we started by disinfecting the skin (antifect® N liquid, Schülke & Mayr GmbH, Norderstedt, Germany). Disinfection was repeated after the skin was removed and scalpels and instruments were changed after each layer. The exposure of the patellofemoral joint was started with the transection of the patellar tendon using new sterile instruments. To avoid alterations of the surfaces the *m*. *extensor digitorum longus* again served as a helpful guiding structure (Fig 1).

**Fig 4:**
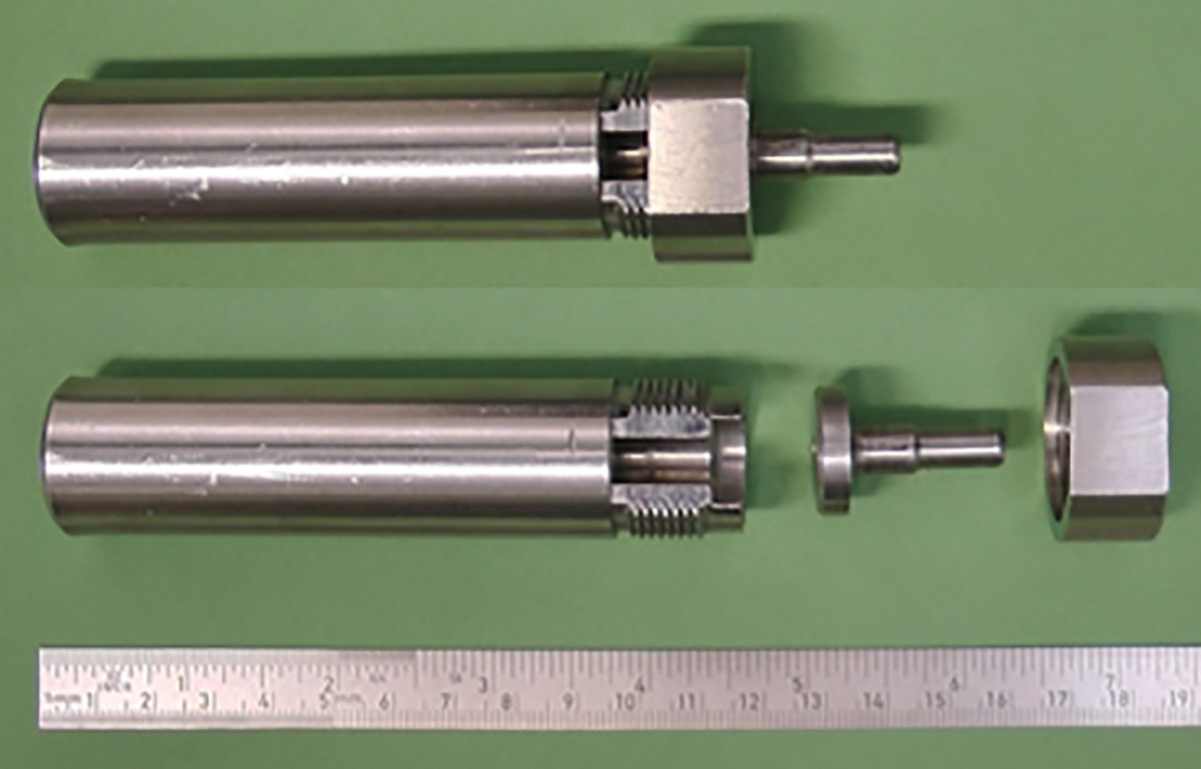
Top: The punching device for the isolation of the osteochondral block. Below: the disassembled hand piece, which makes the sample accessible.

We isolated the samples as osteochondral plugs with a diameter of 5 mm from the trochlea with tools described previously [17]. In addition, we had designed and manufactured another tool, allowing us to also isolate osteochondral plugs from the condyles. We used a punch which was driven by hammer strokes and that enabled us to handle the osteochondral plugs without touching its surfaces (Fig 4).

Eighteen locations were identified as potential sources for “inner” controls in one knee: 6 beside the defects, one was the remaining trochlea *in toto*, 3 from each condyle, and the remains of the condyles, the medial and lateral tibia plateau (n = 2) and the patella.

In the NAT group we punched out the samples from the corresponding regions, so corresponding samples could be identified and harvested (Fig 1).

For histological analysis we used a punch, 10 mm in diameter (inner diameter), to punch out the regenerate (6 mm in diameter) in the middle together with the surrounding tissue. Thus, the analysis of the junction of the regenerated tissue in the defect with the adjacent natural cartilage should become possible in cross sections, according to [34] and [35].

Cartilage and surrounding areas were kept moist continuously with sterile (if needed according to the protocol) PBS. When sterile samples were needed from the trochlea we used sterile instruments and fixation devices [17]. After punching out the plugs, sterility was monitored using swabs at the extraction point of the trochlea [17].

The isolated osteochondral plugs were photographed (Canon EOS 7D; Canon Deutschland GmbH, Krefeld, Germany) fixated on a camera stand [17].

### Isolation of chondrocytes

We isolated the chondrocytes from the articular cartilage tissue of the defects of animals from the E24w and E48w groups. After removal the chondrocytes were processed according to [36]. Then the chondrocytes were counted, the viability was determined by trypan blue exclusion (Sigma-Aldrich, Munich, Germany) and cells were stored in liquid nitrogen.

### Storage and shipping

For stabilization of the tissue for histological analyses the osteochondral plugs were stored in a buffered 4% formaline solution (37% formalin solution, Carl Roth GmbH + Co. KG, Karlsruhe, Germany); diluted with phosphate buffered saline (PBS).

If gene expression analyses were required, the samples were frozen immediately in liquid nitrogen (AirLiquide Deutschland, Ludwigshafen, Germany). For further shipping the samples were packed in layers of dry ice (AirLiquide Deutschland, Ludwigshafen, Germany).

In the case that samples were to be examined in biomechanical tests we put the cylindrically shaped sample into a sleeve (silicon tube with an inner diameter of 5 mm and a wall thickness of 0.5mm (DSG-Canusa, Rheinbach, Deutschland)). They were cut along the rotation axis to a length of approx. 15–18mm. The sleeve was washed and sterilized if needed and then placed into an Eppendorf tube (Greiner Bio-One GmbH, Frickenhausen, Germany) in such a manner that the bony part of the sample was clamped over the edge of the sleeve by the conical part of the Eppendorf tube when it was closed with the cap. Before closing, DMEM (Dulbeccos modified eagle medium, GE Healthcare Europe GmbH, Freiburg, Germany) stabilized with 1% DMSO (Sigma-Aldrich Chemie Gmbh, Munich, Germany) or PBS if required was added and the whole Eppendorf tube was frozen at -20°C before transport.

### Primary stability of the implants

We checked the records and photographs we took, in particular those taken after opening the joints to investigate the primary stability of the implants. The evaluation was done by two observers (MS, GR) according to a 4 point scoring system: filled, almost filled, almost empty, and empty. We were able to check 40 defects, which were treated with matrices with and without cells from the 6 animals which had to be killed or died before the scheduled killing time, and from a pilot animal. The healing time of the assessed animals ranged from 8 days to 92 days (~ 13 weeks).

### Records and lists

We performed protocols for each surgery including premedication and intubation, anesthesia, housing, killing, necropsies, harvesting of samples and other documentation about for example severe events like sudden death. Based on these reports we were able to get an overview over complications and other events which occurred during the operations and the healing periods. Records, data or x-rays were not complete, in some cases also the histories of the animals (missing n = 8). If so, an unbalanced number of cases can result. The assessable numbers are indicated in the results or elsewhere according to the ARRIVE guidelines [21].

The procedure for harvesting the samples was prepared and documented separately for each joint. The shipping of the samples to another place was recorded for each dispatch.

### Radiographic evaluation

The X-rays were blinded and analyzed according to Kellgren and Lawrence (Kellgren and Lawrence 1957) [37] by a senior physician (FBl).

### Macroscopic evaluation

We (MS, GR) evaluated the knee joints macroscopically according to Little et al. [35] and Cook et al. [38] using the photographs that were taken after opening the joints. We also addressed hypertrophic reactions of the cartilage.

### Histologic evaluations

The regenerated tissue in the defects was analyzed histologically with an adapted scoring system according to O´Driscoll et al. [39] after staining with Safranin—O in a blinded fashion (WR, FB, BS, MS). The tissue beneath the defect areas was analyzed (BS, MS) separately according to Little et al. [35] evaluating the condition of the surrounding articular cartilage in terms of degenerative changes. The articular cartilage of the condyles (BS, MS) was checked in the same way.

### Statistics

The EXCEL program (Microsoft Office Professional Plus 2010, Microsoft Deutschland GmbH, Munich, Germany) was used to perform the descriptive analyses of the collected data. Box and whiskers plots were created using the Origin 8.6.0G software (OriginLab Corporation, Northampton, USA).

### Randomization and blinding of the samples

The study design is applicable to up to 8 different types of analysis and 10 animals were needed for a certain type of analysis according to the sample size calculation. Thus, 2 more allocations had to be performed per group. This problem was overcome by randomly allocating a pattern of distribution to the 2 remaining animals. Previously a table was created for 8 “animals” with a selection of samples thus avoiding repetition. This resulted in a template of distribution of the samples in the animals. 2 of the 8 “animals” were chosen by lot and with them the matrix was randomly expanded to 10 animals. When the animals were scheduled to be killed, each animal was allocated to one of the 10 animals in the template by lot again.

About ¼ of the samples were identified by a four digit number (animal number) together with a 2 digit letter (side and location). Later on, we assigned each of the remaining ¾ of the samples a four digits—combination of hexadecimal numbers and letters which were randomly created for each sample using a custom-made program with the LabView® 2011 software (National Instruments Corporation, Austin, Texas, USA). Thus, a blinding regime was applied. For un-blinding the samples a table was created with the blind numbers of the samples, the ID number of the animal, the location of the defect and the type of group.

### Sample size calculation

The sample size calculation for the number of animals which had to be included in the present study was based on a one sample t-test with a significance level of 5% and power of 70, 80 or 90% using the SAS/STAT 9.22 (SAS, Cary, NC, USA) software.

The sample size calculation performed for the histologic analysis individually revealed a need of 10 samples for the control and the treated group with an expected power of 80%.

### Analysis of the regenerates

The results of the scoring of the regenerates according to O’Driscoll [39] were analyzed with the Wilcoxon rank sum test. We compared the NAT group with each of the other groups. Then the difference between the E24w and the E48 group was tested. Finally, the E24w group was compared with the M24w and the MC24w groups and, analogously, the E48w group was compared with the M48w group and the MC48w group.

The effect size according to Cohen [25] was calculated by dividing the difference of both compared mean values by the pooled variance.

### Analysis of the X—rays

The radiographic scoring was tested with the Wilcoxon rank sum test. We determined the differences between the NAT group and the treated groups (M24w, MC24w, E24w, M48w, MC48w and the E48w) regarding the complete joint, the patello–femoral and the tibia–femoral joints. Bonferroni correction was carried out and alpha value was set at 0.0083.

### Analysis of the cartilage of the trochlea and the condyles and macroscopic scoring

The histologic scoring according to Little et al. [35] of the cartilage of the areas of the trochleae adjacent to the defect, the cartilage of the condyles and the results of the macroscopic scoring were tested with the GLM procedure and also post hoc using Dunnett´s test. Each of the treated groups was compared with the NAT group.

In addition, we compared the condition of the cartilage of the trochleae and the cartilage of the condyles using the t-test procedure for each group.

### Correlation between histological scoring, age, and weight

We looked for correlations between age, weight and weight gain and the histological conditions of the cartilage of the trochleae and the condyles respectively. We used the PROC CORR procedure in SAS in order to assess the correlation coefficient according to Spearman.

### Biometrical planning

When, as in the present study more than 1 parameter was to be addressed, it was desirable to create a tool, for a sample size estimation of a study based on likely ES values and the desired power [25].

**Fig 5:**
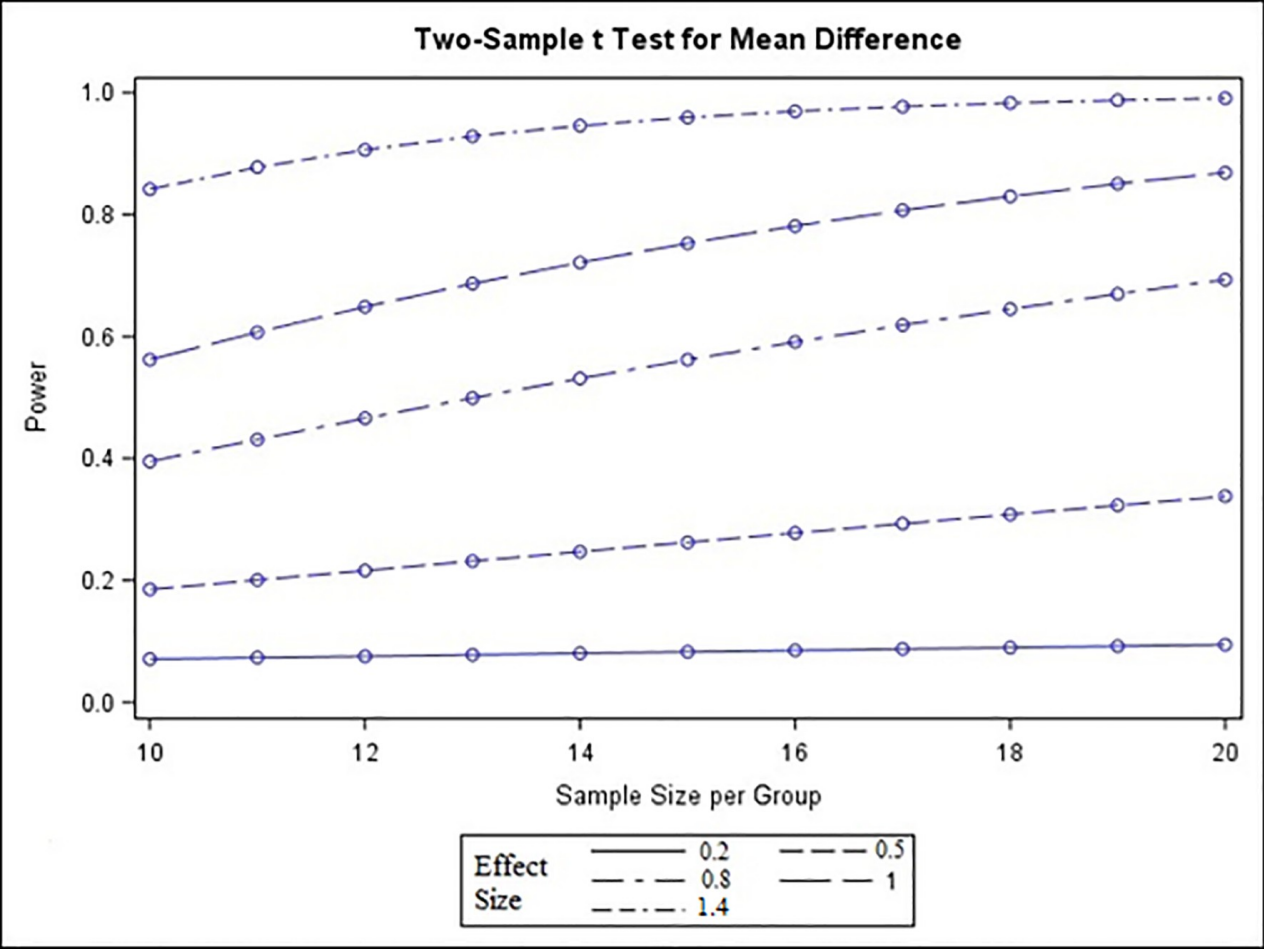
The graph shows the dependency between the sample size and the power, based on 5 different effect sizes (ES). Note: Level of significance α = 0.05 for a two sided t test. The graph shows that an increase of the sample size might not considerably enhance the power, whereas the ES seems to have an important impact on the power.

A graph shows power (y-axis) vs. sample size (x-axis) for 5 different effect sizes for the 2 sample t–test for mean differences (Fig 5). The statistical programming was performed using the SAS 9.3 program (SAS, Cary, NC, USA). The power calculations used the “proc power” procedure for statistical and graphical output.

According to the sample size calculation 10 animals per group were necessary in the presented study.

## Results

### Harvesting

Harvesting of the samples and the shipment to and from the company (Fa. Amedrix) worked well. However, the harvested cartilage material was shipped in a receptacle containing antibiotics (Gentamycin) as the recipient found contamination in the medium after delivery in the pilot phase.

According to the scheduled observation times of 24 and 48 weeks respectively, the animals were killed at an interval of ± 3 days. Thus, the protocol was followed where the correct gathering of samples was concerned.

We harvested 1,837 samples in total from 70 animals (n = 70). 560 of those samples of regenerates and natural cartilage were taken from the defect areas (NAT group), the drop outs (see below) included. 1,277 samples were taken as “inner” controls from other regions of the joint, covered with articular cartilage: a maximum of 3 locations on one facet of the trochlea were identified as potential areas for the “inner” controls but it was only possible to punch out two. Three regions on a condyle were identified, but generally only 2 samples were taken. Remnants of the trochleae, the condyles and the patellae were stored.

Thus, all regenerates (from defect areas) and the double number of samples serving as “inner” controls were isolated.

The menisci were isolated respecting medial and lateral. In addition, blood samples were taken.

### Animals and recovery

In total 7 GMPs more than planned had to be operated in the study, resulting in a failure rate of 9% (7/77).

Two animals served as pilot animals with a standing time of 14 days. The procedure was tolerated well by the animals.

Most of the animals showed abnormalities of the gait pattern for only 1 day after the 1^st^ operation. Further abnormalities were detected for an additional day in 3 cases and in 1 case abnormalities arose at the 4^th^ day once only.

Thus, most animals needed carprofen on the first day after surgery, in two cases no painkiller was needed. Carprofen had to be administered on day 2, 3, 4 or 6 as well. The administration of a stronger painkiller was not necessary.

The gait patterns of all animals were inconspicuous before the 2^nd^ operation.

Most of the animals showed inconspicuous gait patterns during the 12 days following the 2^nd^ operation, approximately 90% (47/52) recovered within the first 8 days day and over 60% (32/52) after the 1^st^ day. Two animals showed normal gait patterns after the 17^th^ and on the 30^th^ day. The observed abnormalities occurred intermittently on different days during the assessed time periods. In 2 cases, on day 165 and 330 respectively, conspicuous gait patterns were detected and treated.

In all cases carprofen was administered on the 1^st^ day after the 2^nd^ operation. Some animals had to be treated several times with carprofen until day 17. Two animals required carprofen on the 30^th^, on the 165^th^ and on the 330^th^ day. Another animal needed carprofen due to an accidental loss of a claw. In few cases buprenorphine had to be administered on day 6 and once from day 5 until 8.

The gait patterns were normal in all cases at the scheduled day of killing.

### Weight gain

The weight of the treated animals increased from 55.9kg (± 10.5kg) to 74.6 kg (±11.6 kg) over 24 weeks and from 49.9 kg (±6.6 kg) to 77.4 kg (±14.3 kg over the observation time of 48 weeks). The weight increase was approx. 33% and 55% respectively. At the end, the weight of the animals in the NAT group 83.4 kg in the mean (±7.61 kg) starting from 61 kg (±7 kg).

### Complications

#### Lost animals

4 animals died, 3 animals had to be killed during the term of the study.

One animal had to be taken out of the study before the 2^nd^ operation as it showed recurrent abscess formation caused by bites during fights.

The day after the 2^nd^ operation we found an animal dead in the cage. The operation was performed without complications according to the protocol. The results of the necropsy were discussed with a pathologist and there was a strong indication that the animal died of a thromboembolic process that affected the lung.

Due to some unmanageable problems in the left knee, another animal had to be killed 8 days after the 2^nd^ operation. The necropsy revealed abscess formation in the soft tissue of the left knee joint, but the inner joint space was inconspicuous.

On days 19, 46 and 60 after the 2^nd^ surgery an animal was found dead in its cage.

The necropsy of one animal revealed pneumonia and abscesses in the lungs after treatment of a respiratory disease.

One animal died of a toxic circulatory failure based on a hemorrhagic colitis with appropriate clinical symptoms and failed therapy.

One animal was lost due to an acute heart failure based on a fresh myocardial infarction with alveolar and interstitial edema on spec, as the tissue showed pronounced autolytic changes. The clinical aspect showed a discoloration of the integument, reduced appetite and fever. The animal died despite appropriate medication. The gait pattern was inconspicuous before death.

One animal had to be killed 92 days after the 2^nd^ operation due to unmanageable suffering showing weight reduction, low temperature (31°C) and a marbled integument. The animal showed symptoms like nervous discoordination, comatose and convulsive spasms. Before that, the animal had shown an unsuspicious lethargy. Despite appropriate medication, it did not recover and we decided to kill the animal. The necropsy revealed an abscess, allocated to the Tympanon as most likely point of origin.

#### Infections

After the 1^st^ operation 3 animals developed an abscess and could not be operated the 2^nd^ time. They were the so- called “drop outs”. One had to be killed due to unmanageable abscess formation with bite marks. Thus, the infection rate following the 1^st^ operation was 6.7% (4/59).

Apart from infectious processes found in those animals that were taken out of the study we identified superficial infections (referred to as “superficial abscesses” (2 / 98 knee joints) and “exudative events” (8/98 knee joints) in 10% of the knee joints (10/98) in the region of the wound after the 2^nd^ operation. They were successfully treated by rinsing them with NaCl solution (B.Braun, Melsungen, Germany) and anointing them with Braunovidon® (B.Braun, Melsungen, Germany)

In one case an abscess formation at the right knee joint 12 days after the 2^nd^ operation was surgically removed, revealing an unaffected inner joint space. The animal recovered within 6 days after a 5 day long antibiotic treatment.

In another case, purulent discharge appeared which originated from a stitch channel after the animal was prepared for the 2^nd^ operation. This was surgically treated as well. We then performed the 2^nd^ operation, using new instruments as planned. Antibiotics were administered for a period of five days.

Positive smear tests were found during the operation in 50% (8/16) of the affected knee joints.

During smear tests were processed, the treatments were done with antibiotics with broad spectrum (Pen-Strep: aniMedica GmbH, Senden-Bösensell, Germany; Enrofloxacin: Baytril ® 2,5%, Bayer AG Leverkusen; Germany; Norfloxacin: Norflox-200, Interchemie, Alendaar, Niederlande) if needed.

Some other abscess—like formations were seen at the right region of the face, the right gluteal region and one at the right leg which healed spontaneously.

At one knee of a pilot animal we have seen a superficial abscess in the region of the patella while preparing the knee after killing. The inner space of the joint was clear without signs of any infection.

#### Further complications

When opening the knee joints for the 2^nd^ operation we found subcutaneous seroma in 87 cases (73%). In 14 of those cases we identified germs during microbial analyses, in one case a staphylococcus aureus was identified. The other swabs revealed facultative pathogen types of germs.10 animals suffered from diarrhea (16.1%) and recovered without further complications. One of those animals belonged to the NAT group.

One animal showed a purulent exudate from the suture, though this is not defined as a surgical site infection according to [40].

One animal developed fever (38.6°C) 29 days after the 2^nd^ operation and was treated successfully (Novalgin® Tropfen, Sanofi-Aventis Deutschland GmbH, Frankfurt am Main, Germany).

With one animal an outer horny part of the claw at the left hind leg was pulled out while transferring the animal to the transportation cage. Pain killers (carprofen) were administered until the animal was able to fully load the leg again (~11 days). The animal showed no abnormal gait afterwards.

#### Operations

During the 2^nd^ operation we noticed a higher tendency for the tissue to bleed and to swell than during the 1^st^ operation, complicating the surgical approach in particular of the most lateral proximal defect area (Fig 1).

The 2^nd^ operation was performed between the 7^th^ and the 15^th^ day after the first operation with a mean of 10.05 days (± 1.9days). The wounds from the 1^st^ operation had healed by that time.

In all cases, a thin membrane formed spontaneously in the defects between the 1^st^ and the 2^nd^ operation [41]. This was removed to restore the shape and the defined volume of the defect as well as to clean the borders of the cartilage tissue and to refresh the bottom part of the defects.

Apart from the tendency to higher bleeding we observed as described (before) with the pilot animals, we detected effusion in the knee joints in 12 cases (6 left, 6 right). In 17 cases synovitis was evident (8 left, 9 right). In 5 cases, both effusion and synovitis developed together in the same joint.

We had to suppress heavy bleedings with Metoprololtartrat (Beloc®, AstraZeneca GmbH, Wedel, Germany) in two animals during the 2^nd^ operation.

The suture material was removed on the 9^th^ day (± 3 days) after the 2^nd^ operation. In 9 cases sedation was necessary to remove the suture material. In several cases, the suture material was removed a bit at a time due to infectious processes (see above).

### Operation times

**Fig 6:**
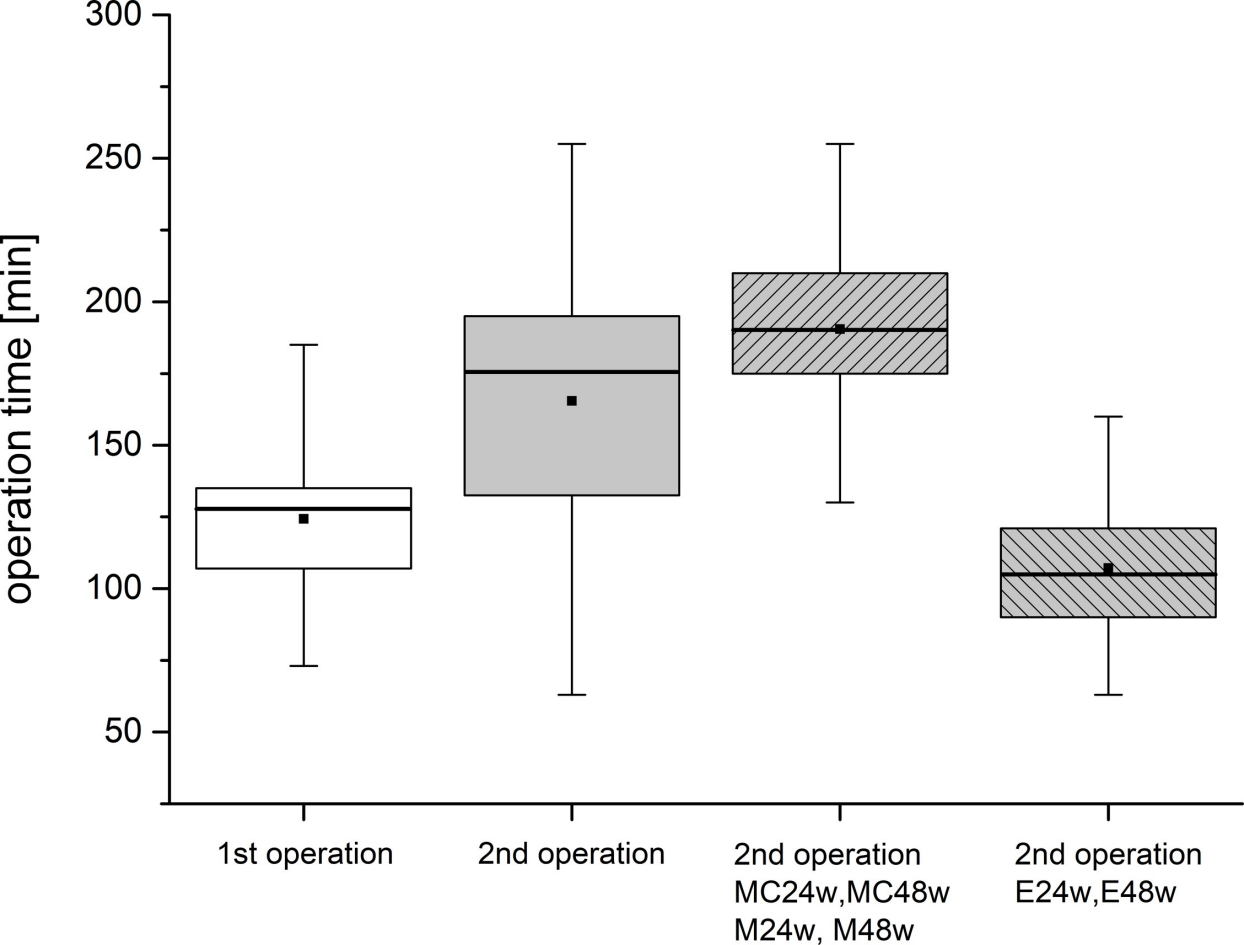
Times of the surgeries calculated for both knee joints together. The data of the 1^st^ and the 2^nd^ operation according to the protocol are included, also those of lost animals. The 2^nd^ operation was more time consuming than the 1^st^ one. The operations during which the implants where set in the groups MC24w, MC48w, M24w and M48w took the longest. The operations in groups E24w and E48w were the shortest as the defects were left empty. Thus it was possible to calculate the net value of setting one implant at approx. 10 minutes.

The 2^nd^ operation when the implants were set (n = 60) took 53% more time than the 1^st^ operation with approximately 124 min calculated per animal (n = 65) (Fig 6). Thus, the implantation of matrices took approximately 80 minutes (83.4 min) per animal, that is to say 10 minutes for the treatment of one defect with an implant.

### Chondrocyte isolation

The volume of the cartilage tissue gained from one defect could be calculated as 14.14 mm^3^ with a defect diameter of 6 mm and 0.5 mm in depth. In 10 cases the number of isolated cells amounted to 60,400 ± 32,400 cells per defect volume. The numbers of cells per tissue mass was 340,000 ± 134,000 per g tissue (n = 7). The viability of cells exceeded 95%.

According to the collaborating company (Fa. Amedrix) the number of isolated chondrocytes was sufficient in order to perform autologous MACT in the MC24w and the MC48w groups.

### Primary stability

According to the manufacturer’s guidelines, the repeated moving of the joint after the implantation of the matrix seemed to be appropriate and feasible for testing the primary stability of the implants. In some cases, the implantation had to be repeated.

The healing time of the assessed knee joints in terms of primary stability in place ranged from 8 days to 92 days (~ 13 weeks) after implantation. We calculated that 92% of the implants remained in place in 40 assessed defects. Two defects, which were located at the proximal position of the lateral facet, were marked as empty and one was marked as almost empty (Fig 1).

### Autopsy

At the end of the scheduled time, each animal was examined in an autopsy. No serious changes to the inner organs were found. In some cases (n = 6) there was a hardening in the retro-peritoneal region and the fatty tissue. The histological examination of one case, which was used as a representative, revealed an older, partly calcifying reactive lipolytic necrosis of fatty tissue.

### Statistics

According to the sample size calculation 10 animals per group seemed to be sufficient in the presented study (Fig 5).

### Histologic outcome of the treatments

**Fig 7:**
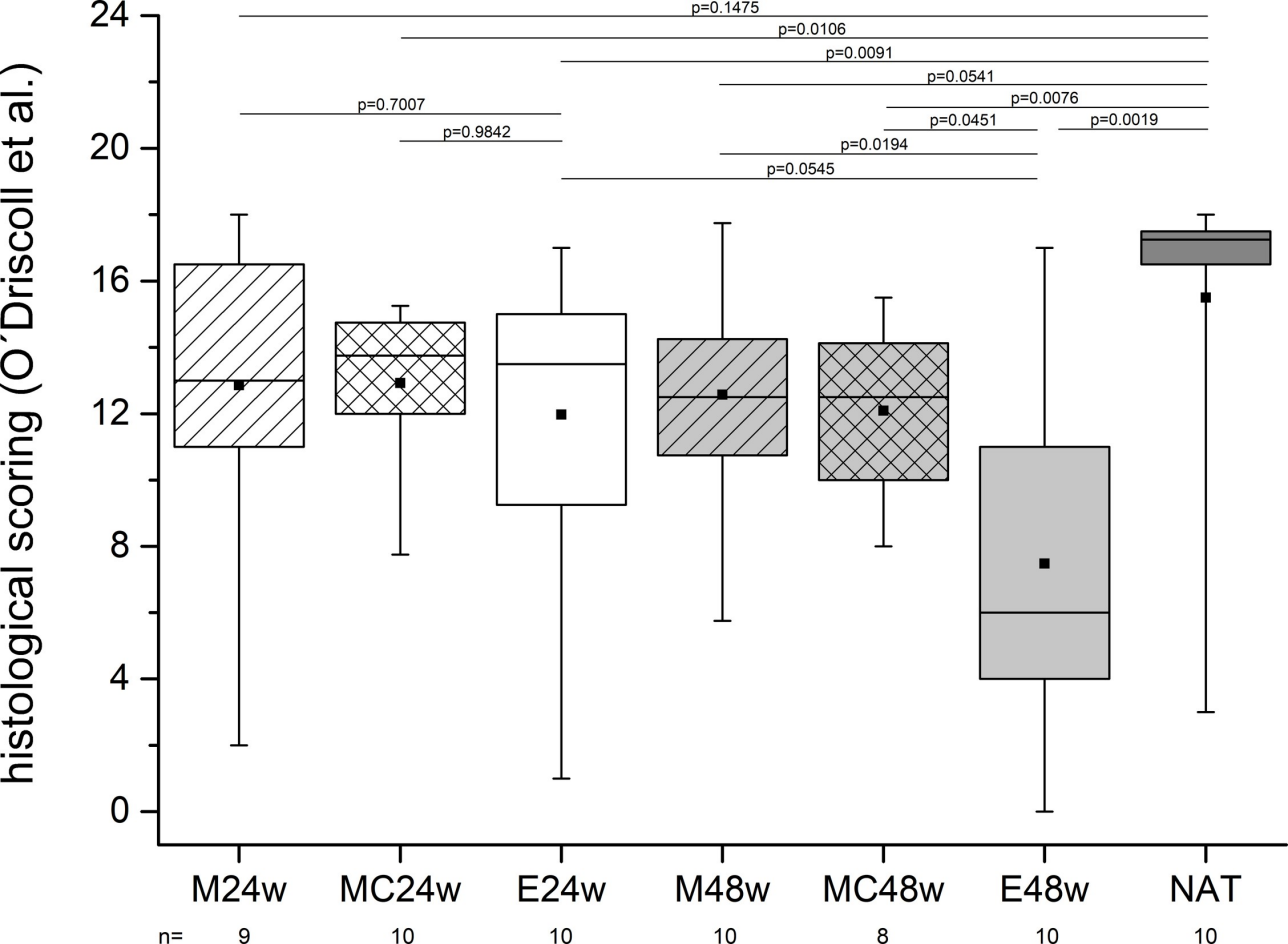
The scoring of the histological specimens revealed the NAT group as the group where the cartilage was in the best condition. The groups treated with matrices without autologous chondrocytes (M24w and M48w) showed no significant differences compared to the NAT group. The groups with the cell laden matrices (MC24w and MC48w) had significant lower scorings. The comparison between the groups treated with implants (M24w, MC24w, M48w and MC48w) and the corresponding groups where the defects were left empty (E24w and E48w) showed significant differences after 48 weeks but not after 24 weeks. Note the wide spread data and the small differences between the mean values in the treated group.

The specimens of the NAT group were identified with one outlier (Fig 7). Significant differences were seen between the NAT group and the E24w, E48w, MC24w and the MC48w groups. The E48w group achieved less scoring points than the E24w group but not significantly. The comparison of the treated groups with empty groups at the same point in time revealed significant differences only after 48 weeks observation time between M48w and E48w (p = 0.0194) and between MC48w and E48w (p = 0.0451).

The effect sizes are listed in Table 2, revealing numbers from 0.18 to 1.64 with the p–values set next to it (Table 2).

**Table 2:** The p—values and ESs of the assessed comparisons between the groups, juxtaposed. The ESs can be ranked according to their values. Thus, a ranking of the effectiveness of a procedure compared with a control is possible. Note that the difference between the E24w group and the E48w group is not significant but reveals a large ES with 0.89. *Vice versa* the difference between the NAT group and the MC24w group is significant but only has a medium ES of 0.7.

group comparison	p-value	Effect Size (ES)	rating
NAT-E48w	0.0019*	1.64	> 0.8
M48w-E48w	0.0194*	1.16	> 0.8
MC48w-E48w	0.0451*	1.1	> 0.8
E24w-E48w	0.0545	0.89	> 0.8
NAT-MC48w	0.0076*	0.88	> 0.8
NAT-E24w	0.0091*	0.73	> 0.8
NAT-M48w	0.0541	0.71	0,5–0,8
NAT-MC24w	0.0106*	0.7	0,5–0,8
NAT-M24w	0.1475	0.55	0,5–0,8
MC24w-E24w	0.9842	0.24	< 0,5
M24w-E24w	0.7007	0.18	< 0,5

The used ranking intervals are determined according to Cohen [26]. Usually, Cohen´s effect measures with < 0.5 are regarded as small, effect measures with values between 0.5 and 0.8 as medium and effect measures > 0.8 as large effect.

* = significant; see Fig 7

One specimen in the NAT group scored very low. This could be explained by the fact that the specimens were examined in a blinded manner.

### Effects on the articular cartilage close to the defects and the knee joints

We evaluated the impact of the treatments on the articular cartilage both adjacent to and further away from the defects in the stifle joint.

### Radiographic evaluation

**Fig 8:**
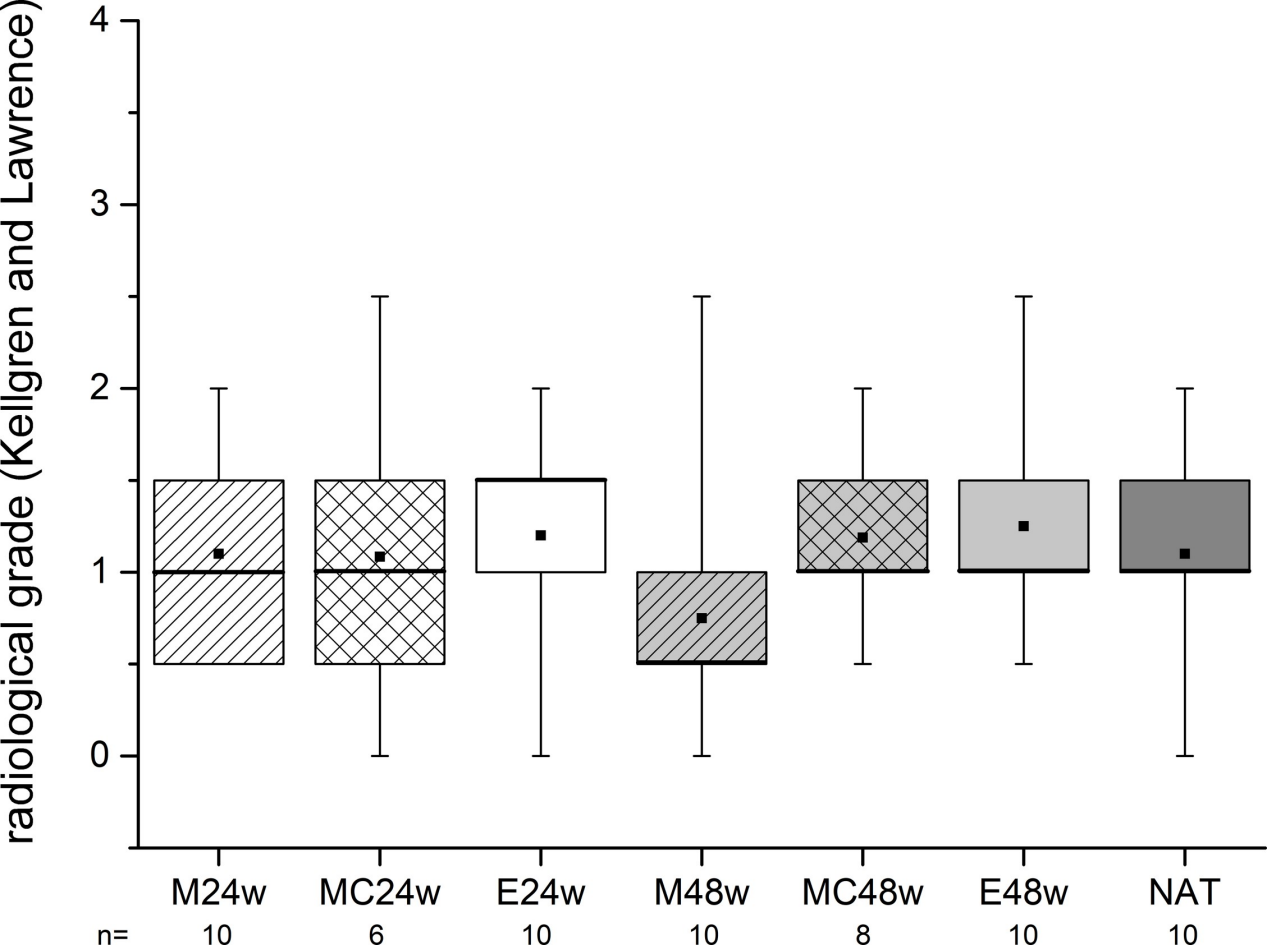
The scoring data of the x–rays revealed no significant differences between the groups in terms of degenerative changes.

The radiographic examination delivered scoring points ranging from 0.75 (± 0.72) to 1.25 (± 0.59) assessing the joint as a whole. We found no significant difference between the NAT group and any of the treatment groups (Fig 8).

### Macroscopic evaluation

**Fig 9:**
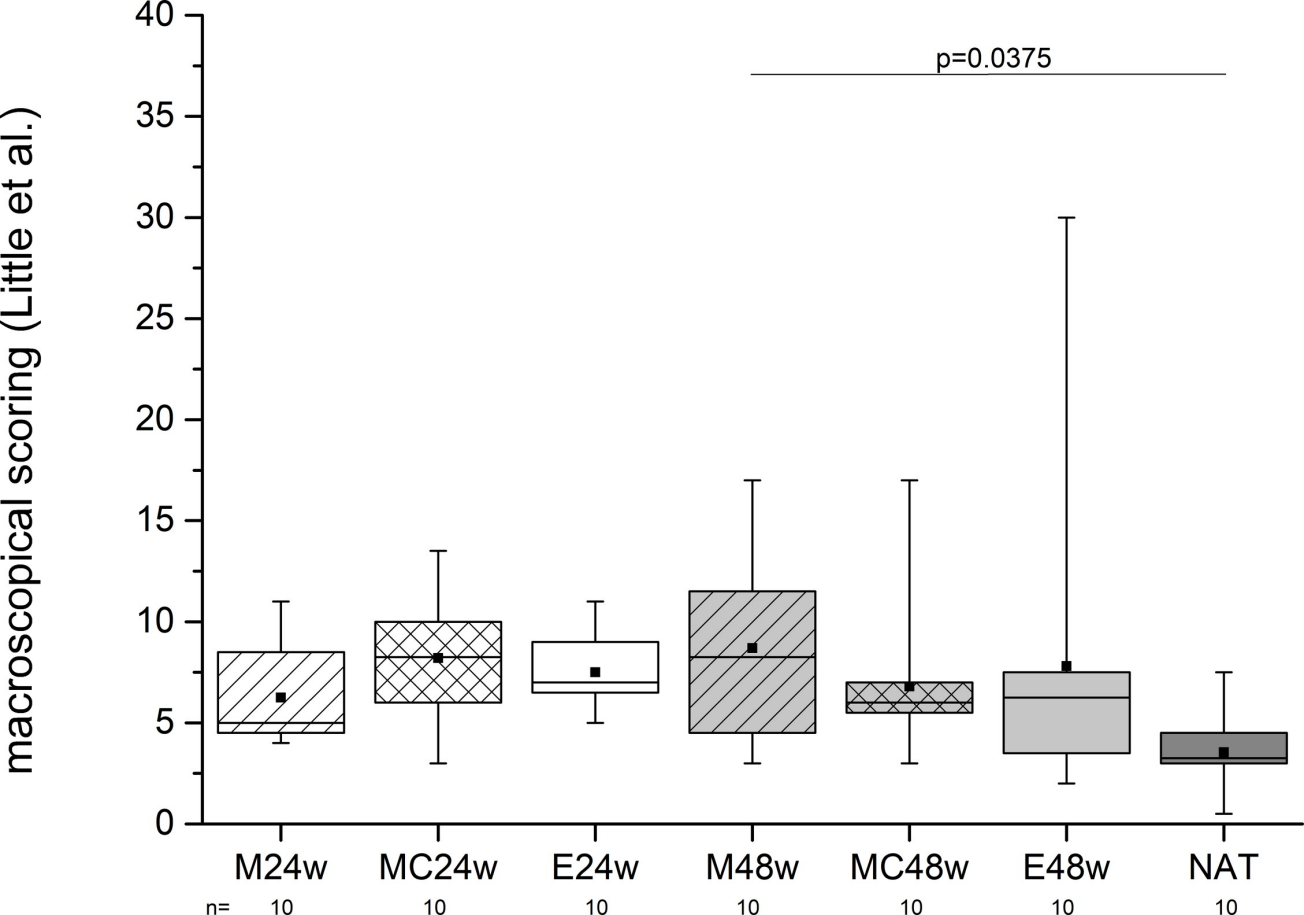
The stifle joints were examined using the photographs taken immediately after the opening of the joints. One significant alteration was detected, but the worst specimen was found after 48 weeks in a knee joint where the defects were left empty (E48w).

We found degenerative changes in the knee joints ranging from 3.55 (±1.79) scoring points to 8.7 (±4.55). The maximal value was 30 in the E48w group. The M48w group showed significantly more points than the NAT group (p = 0.0375) (Fig 9).

### Histologic evaluation (trochleae)

**Fig 10:**
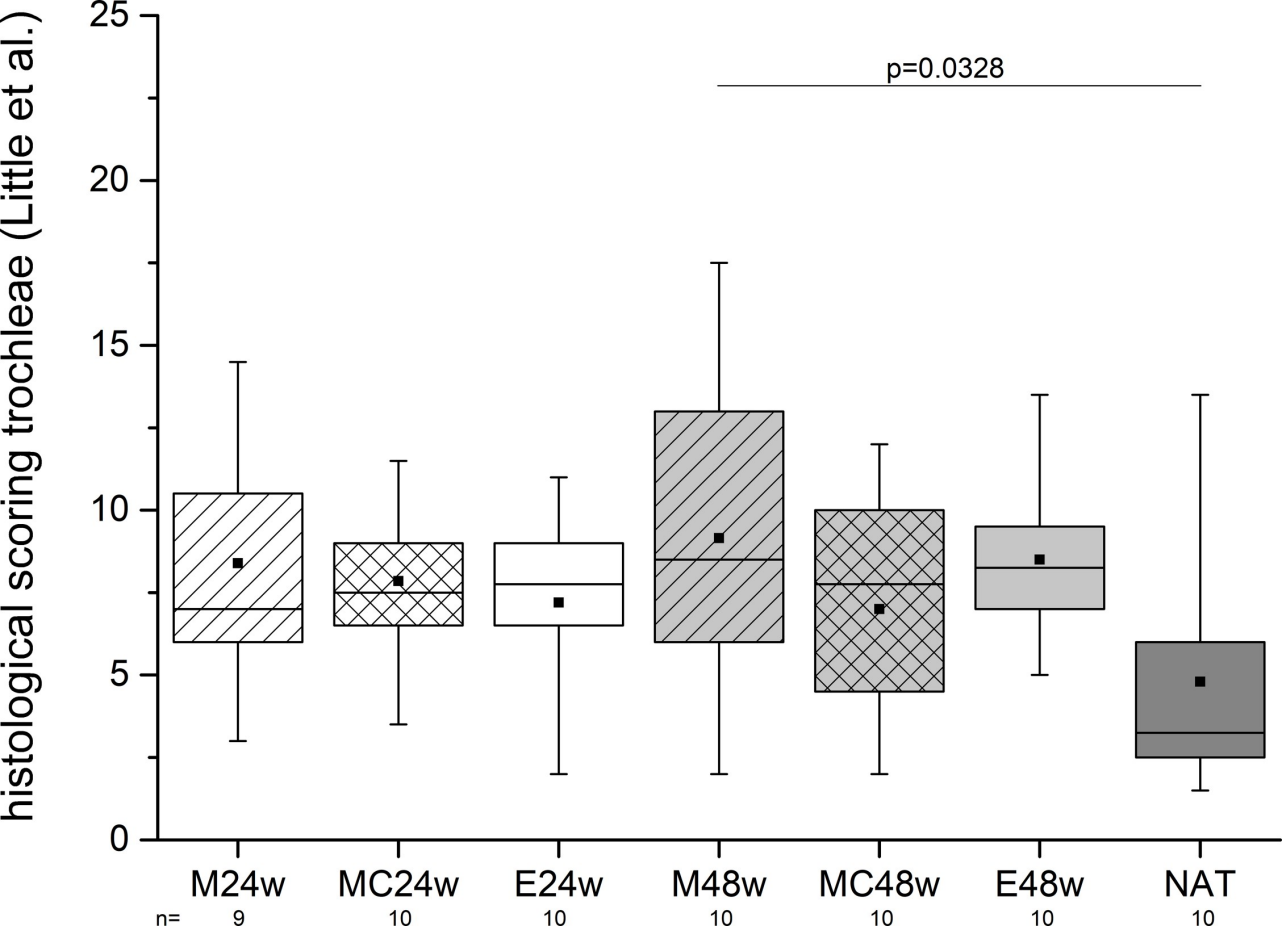
The articular cartilage was examined near the defects. Even the NAT group showed some degenerative changes. Comparing the condition of the cartilage, the tissue in the M48w group was in a worse condition than that in the NAT group.

The articular cartilage adjacent to the defect areas was significantly affected histologically in one case (M48w group). The NAT group showed the lowest number of scoring points here (4.8 ± 3.38) (Fig 10).

### Histologic evaluation (condyles)

**Fig 11:**
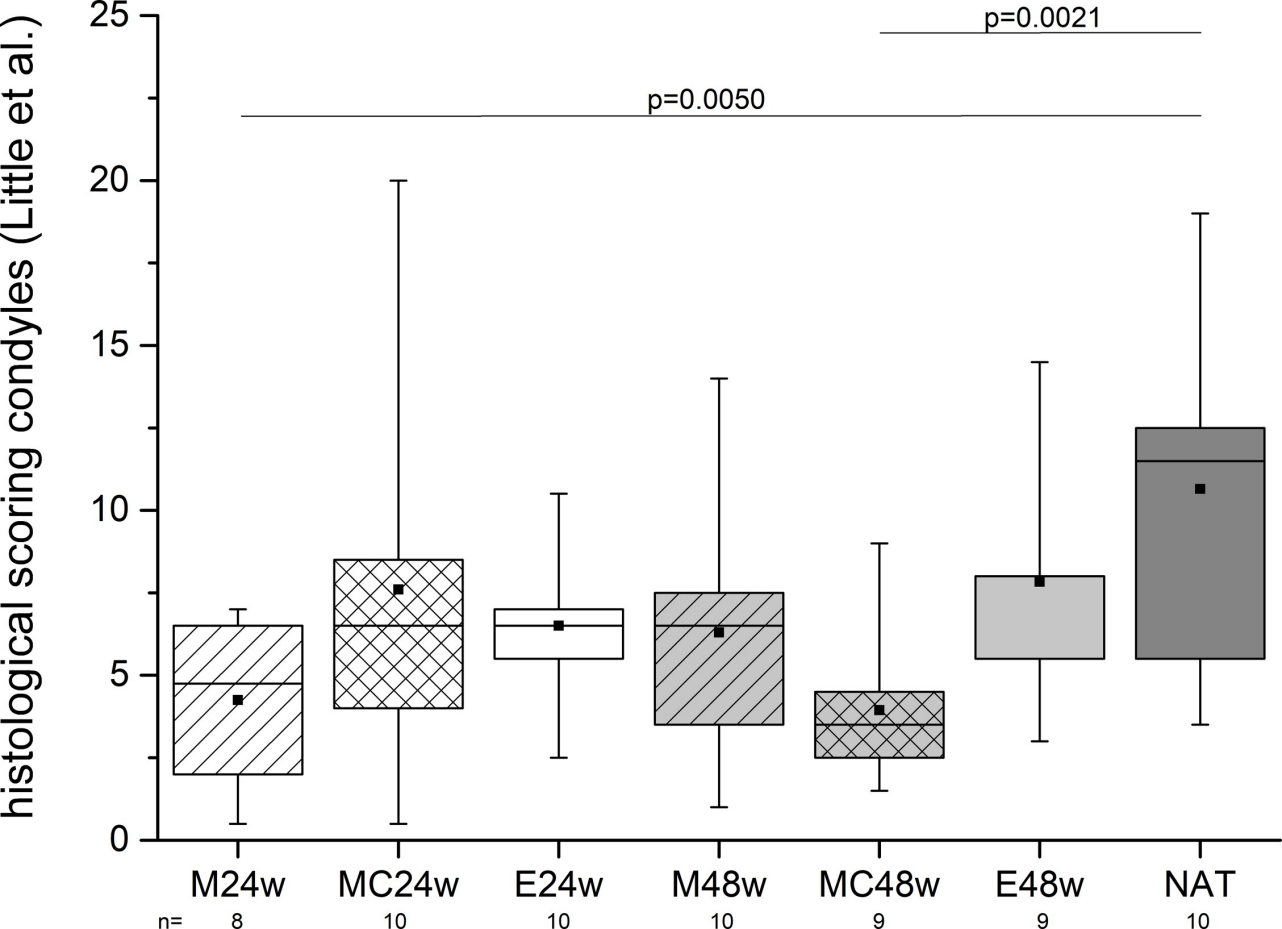
The condyles were examined as remote regions to the trochleae were the defects were created. Surprisingly, the NAT group revealed the worst condition of the articular cartilage of all groups. Two treated groups were actually significantly better. The results show that in this age group the GM can develop degenerative changes at the condyles rather than at the trochleae (Fig 10).

The articular cartilage of the condyles revealed the highest value in the NAT group in terms of degenerative changes with significant differences to the M24w and the MC48w groups. The articular cartilage of the condyles in the treated groups revealed a better condition than that found in the NAT group (Fig 11).

### Comparison between trochleae and condyles

The pairwise comparison of the condition of the cartilage of the condyles and the cartilage of the trochleae showed a significant difference (p = 0.002) in the NAT group with a higher scoring of the condyles according to Little et al. [35].

### Correlations: histological condition, age, weight, weight gain

We found a significant correlation between the age of the animals and the histological condition of the cartilage of the trochlea according to Little et al. [35] with p = 0.0483 and ρ = -0.63554 in the MC24w group. Another correlation was seen between the weight of the animals and the condition of the cartilage of the trochlea (p = 0.0038; ρ = -0.81850). We did not find any correlation between the histological condition of the cartilage and the weight gain, neither where the trochlea was concerned nor the condyles.

### Hypertrophic reactions

We identified 9 hypertrophic reactions in the operated knee joints (15/112) after 24 weeks and 6 hypertrophic reactions after 48 weeks. In the groups where the defects were left empty, 3 hypertrophic reactions were seen after 24 weeks (E24w) and 1 after 48 weeks (E48w) respectively. 3 months after surgery, we were able to find a hypertrophic phenomenon in the right knee joint at the lateral distal defect area of an animal that was initially included in the MC48w group, and had to be killed 92 days after treatment.

## Discussion

The assessment of a new method for regenerative articular cartilage therapy is challenging as no gold standard exists, which may accurately and unequivocally judge the quality of the recreated tissue as suitable or not. Several test procedures are available, like biomechanical, biochemical or molecular-biological; thus, one may get the idea to evaluate a new treatment and regenerate samples in particular with several types of analyses. Hence, the preclinical evaluation becomes vital, as human trials are limited for scientific evaluation.

With this paper we present a protocol of a large animal model which is able to produce up to 8 regenerates in one animal plus several types of control samples (also in a high number). Thus, the presentation of our findings in this study completes our recently published study on newly developed instruments to standardize preclinical tests for articular cartilage regeneration therapies [17].

Several publication describe the animal model we used [14, 16, 42, 43]. We saw some advantages in this model, like the comparability of the swine with the situation in humans [14, 27, 44, 45], the surgical procedure, the biomechanical situation in the trochlea [46], the rather flat shape of the trochlea [42] and the fact that this model is suitable for the creation of several defects in one knee joint.

The groups E24w and E48w (Table 1) could be seen as control groups in terms of critical defect size, determining if spontaneous and endogenous regeneration of cartilage can occur in empty defects in the GM ([27] cite [14]). The groups in which the animals received implants without cells (M48w and M24w) can serve as control groups for those with seeded cells (MC24w and MC48w) or even as *verum* groups by themselves in terms of a cell free matrix used for therapy.

As both knee joints of one individual are involved, the discussion about asymmetrical load bearing or the occurrence of relieving one limb is void. Thus, the quality of all gathered samples will be comparable with regard to the site of origin, treated or not, the weight bearing and the individual animal.

Only one type of treatment was used in both knee joints of one animal. By doing so, interactions between different types of treatments within one joint could be excluded as well as systemic influences between both knee joints. This may become important, if anabolic substances are part of an implant as reported by Sumner et al. [47].

### Number and size of defects and operation procedures

The creation of more than one defect in large animals was described in several studies [13, 16, 27, 48]. It is trivial to state that the larger the animal the larger the defect that can be created for delivering regenerated tissue in greater dimensions [12] [19]. It is important to keep in mind that larger samples of cartilage tissue are more suitable for biomechanical testing due to their appropriate dimension [11, 19, 49, 50]. Mainil-Varlet et al. [16] created 6–8 defects, 4 mm in diameter, in the patella groove of Yucatan minipigs. Blanke et al. [15] reported a number of 6 defects of 5 mm in diameter in one trochlea of minipigs. Sosio et al. [41] also created 6 defects but with 6 mm in diameter in the trochlear groove of the right knees of 4 months old Large–White pigs. Frequently, defects were set in both joints of one animal [27, 48]; if not, the contralateral side was used as untreated control [18]. Fisher et al. [48] and Kim et al. [13] reported 8 defects in both trochleae of Yucatan minipigs, 4 on each side with a diameter of 4 mm.

Five defects were set in one stifle joint of pigs at both sides in a standardized way [51] by Hembry and coworkers [52], but rectangular in shape (0.5 mm wide, 0.5 mm deep, and 15 mm long). Two were positioned at each facet of the trochlea and one was placed at the medial femoral condyle. As to the latter, our concern is that there are no identical starting points for all defects in this model. Therefore, we restricted the area for setting defects to both facets of the trochlea. In contrast to [52] we prefer defects cylindrical in shape as in our opinion the setting of those defects can be performed in a highly reproducible manner using the instruments as described by our working group [17]. The defect area for all of the 4 defects is approx. 28 mm^2^ and therefore more than 3 times larger than the total defect area described by [52]. Griffin et al. [12] created two large cartilage defects with approximately 177 mm^2^ each (15 mm diameter) in one trochlea of the knee of one horse and harvested 3 cylindrical plugs for biomechanical analyses and half of the defect—area for histological assessment after 1 year [12]. Thus, a large defect offers the possibility to create some samples for different analyses, provided each section of the regenerated tissue is of the same quality. However, the use of horses may be restricted to few research centers.

We created defects with a diameter of 6 mm *in vivo* and harvested samples with a diameter of 5 mm with the intension of separating material of regenerated cartilage from natural cartilage tissue. This procedure seemed to be the best for biomechanical tests like compression [11, 19, 53, 54] or tribological analyses [49]. The disadvantage is that the surrounding natural cartilage tissue is lost for analysis of the interface between the regenerate and the original tissue by doing so. Therefore, we harvested the samples for histological analyses in a larger dimension to ensure that both, the regenerate and the natural cartilage, are taken from one specimen in order to enable the use of an appropriate scoring system [34].

### 1^st^ and 2^nd^ operation

We timed the 2^nd^ operation within a time frame of about 14 days after the 1^st^ operation. The type of implant used (Fa. Amedrix, Esslingen, Germany) made this procedure possible, as the processing for re-implantation of the cells took some days.

The regeneration tissue at the bottom of the defect was easily removed but we assume that the longer the time between the 1^st^ and 2^nd^ operation the greater the spontaneous regeneration process, resulting in a more or less developed scar that would have to be removed for the correct implantation of the matrix. However, the spontaneous healing process was interrupted by the presented procedure. Sosio et al. [41] described a repairing tissue which developed between the 1^st^ and the 2^nd^ operation, which they performed 3 weeks after the 1^st^ one. They also harvested chondrocytes from 6 defect locations, 6 mm in diameter, in the trochlear groove for the treatment of osteochondral lesions in the same locations in 4 month old Large—White pigs [41].

Similar to the procedure in the present study, [55] reported the harvesting of autologous cartilage material from the lateral femur condyles of goats in order to re-implant the expanded chondrocytes 4 weeks later in the same defect. Lind et al. [56] re-implanted the chondrocytes within fibrin-hydrogel and MPEG-PLGA scaffolds in the same defects in goats, 4 weeks after the 1^st^ surgery. The time between the creation of the defects and the implantation of the autologous chondrocytes seeded in a I/III collagen membrane was 6 weeks with [57]. The defects were created in the trochlea and the medial femoral condyle in sheep, whereas the cartilage material, from which the chondrocytes were isolated, came from the non-weight bearing lateral supracondylar area [57].

### Welfare

The involvement of pilot animals can be recommended. Despite the fact that many technical procedures can be developed and refined using specimens taken from cadavers, other questions could only be answered by operating on the living creature. Here, it is possible to take into account the hygienic environment or the behavior of the parameters like the blood flow and inflammation response. The surgical approach was slightly different from *in vitro*, especially at the 2^nd^ operation. The pilot studies also showed us, that it was possible to set the implants shortly after setting the defects, from which the cells were harvested. We also learned to store the removed tissue in antibiotics for shipping to the manufacturer. The work on pilot animals, which were not included in the trial, was essential to find the final protocol, which was rigorously applied during the whole trial. Thus, an important part in the reproducibility of the presented trial is based on the use of pilot animals. An expedient use of pilot animals is described also by [16], who analyzed the fixation of the implants or [13] who also examined short time effects of a growth factor and the degradation of the scaffold after 2 and 6 weeks.

As expected, the time of recovery after the 1^st^ operation was shorter than after the 2^nd^ operation. Where an abnormality of gait patterns is concerned, the longest time period was the 4^th^ day after the 1^st^ operation. The resection of parts of the fat pad seemed not to have induced further distress. Caminal et al. [30] and Murray and Fleming [58] reported a similar procedure.

The recovery after the 2^nd^ operation was comparable to the findings of [42]. We can state that, in the present study, distress of the animals after the 2^nd^ operation was similar to the distress felt by minipigs after the setting of osteochondral defects in the medial femoral condyles with a postoperative time of recovery of up to 7–10 days. Murray and Fleming reported a full weight bearing of Yucatan mini-pigs within 48 to 72 hours after ACL reconstruction [58]. An explanation for the longer time of recovery in the present study could be that the duration of the 2^nd^ operation was about 1 hour longer when implantations were performed than the time needed for the 1^st^ operation.

The higher distress after the 2nd surgery can also be deduced from the need of strong pain killers in some cases and from a longer period of pain medication.

However, the animals recovered well before the 2^nd^ operation and showed a healthy condition until the scheduled time of killing.

### Weight gain

The longer period of observation (48 weeks) brought about a higher weight gain than the shorter one (24 weeks). The animals reached body weights comparable with those reported by [59] in a study with obese 4–6 years old GMs after ovarectomy. Schinhan et al. [18] reported a weight loss of 3.7% of the animals in their OA model after an observation time of 6 and 12 weeks. This is different to the present study. However, one has to take into consideration that we did not perform an OA model which would have put a strain on the animals. That could have led to weight loss due to some distress resulting from the treatment. Another reason for the weight gain may be the low activity level the animals showed during the observation time after surgery. The observed weight gain of the treated animals was an important reason to keep the animals of the NAT group in the same environment and conditions as the operated pigs. The fact that the NAT group was not observed for 48 weeks could be seen as a disadvantage. However, we noted that there was a 32% weight gain in the groups that were treated over a period of 24 weeks groups and 62% in those that were treated over a period of 48 weeks. There was a 38% weight gain in the NAT group therefore close to the groups of treated animals. However, we think that the weight gain is a strong indication for the healthy condition of the animals during the healing period.

### Number of animals

A major factor that has to be considered when performing a study like the one we presented is the large number of animals needed. One also has to find a breeder who is able to deliver a herd of 70 animals all at once. Another important factor is the capacity of the facilities, the number of the operation theaters, the staff and the time frame of nearly one year needed for the follow up treatment etc. The complexity of the logistics is increased when animals have to be housed separate as not all animals tolerate the housing in groups [10]. This can lead to serious complications even to the loss of an animal (see complications). However, the animals should not be separated completely [10] to avoid stress, as recommended by [60]. Thus, we had to perform the operations over a period of 32 months and in groups of 10 animals each, according to their grouping (Table 1). This may be seen as a disadvantage of the study but a random regime of treatment would have been hardly feasible and complications in terms of protocol deviations (confusion) would have become more likely. This can happen in complex studies that include a huge number of animals [58]. The huge number of animals we needed made it necessary to obtain the GM from two breeders; one is located in Germany, the second in Denmark. According to [27] there are 4 different breeding facilities of GM in the word: Denmark, Germany, the US and Japan [27]. Simianer and Köhn [61] reported on a small but unavoidable genetic differentiation between the German and the Danish GM populations, but it was without statistical significance. However, the availability of a huge number of GM has to be considered and breeders should be involved as early as possible, as supply-bottlenecks could arise.

### Age

One aspect in the articular cartilage research is the skeletal maturity of the species included. In the research maturity is necessary if one wants to avoid the confounder of young tissue with a higher proliferation rate of the chondrocytes and production of extracellular matrix [27, 62–65]. The animals included in the present study were at least 24 months old and skeletally mature. Some literature referred to an age of 18 months [27]. However, spontaneous degenerative changes of the articular cartilage cannot be excluded in the swine [66], as shown in our results (Fig 11). Additionally, we did not see a disadvantage in the use of older animals in our study as the results are more comparable to the human situation, where older individuals also suffer from changes in the articular cartilage [67, 68].

### Samples

As the borders of the defects were still identifiable, we were able to harvest the samples according to the protocol. They measured 5 mm in diameter and were therefore smaller by 1 mm in diameter than the defect which measured 6 mm precisely. For the histological analyses the procedure was different. We included the surrounding tissue of the defect, thus making it possible to apply an appropriate scoring system [39]. Apart from those samples with the regenerated tissue, we were able to collect a large number of controls. However, the sample number was limited as we left the lateral and medial tibia-plateaus intact. These could have delivered several more samples for detailed analyses of the articular cartilage as described by [69, 70].

### NAT Group and “external” controls

The inclusion of the untreated control group (NAT) made it possible to analyze the impact of the surgical treatment [20]. In the NAT group we defined the same individual locations in the knee joints for taking samples as in the other groups. We had to respect the locations where the defects were set as well as those where the “inner” controls were taken, e.g. from a condyle (see below). A further advantage of the inclusion of the NAT group is the possibility of saving animals (see below: the 3Rs).

### Primary stability

We checked the stability of the implants in the defects immediately after implantation by moving the joint several times, as recommended by the manufacturer. For analyzing the stability of the implants later on we checked knee joints of those animals that did not stay alive up to 24 weeks. We inspected these joints as we knew that we would not be able to determine macroscopically if the tissue found after 24 weeks or even after 48 weeks was a spontaneous regeneration or a regenerated caused by the implant. However, we estimated that more than 90% of the implants would remain in situ. We recommend that the most proximally located defects need to carefully implanted.

### Complications

After killing the animals we performed necropsies to identify other complications that could be caused by the treatment, in particular the treatment with cell loaded implants. The identification of cell migration as ectopic tissue is required to determine the potential of migration far away from the “site of administration” [71]. The procedure which we performed was presumably not absolutely appropriate to fulfill the safety requirements [71, 72] but we intended to avoid overlooking severe adverse events which could be traced to the treatment. In some cases we found an induration of the retroperitoneal fat which was assessed by the pathologist and interpreted as unsuspicious. However a sound risk prediction of a cell based therapy needs a more sophisticated protocol as we were able to apply in the presented study [30, 73].

### Lost animals

Unfortunately we lost 7 animals in the study. Spontaneous death seems to be a seldom event in a planned study as we did not find many reports in literature we have checked.

Apart from one case amongst all lost animals we were not able to correlate the death or the illness of the animals with the surgical treatment. Wang et al. [43] lost 3 pigs due to wound infections.

We learnt to be quite vigilant regarding the social behavior of the animals as we lost one due to the consequences of fights with other animals [10].

In another case the welfare of one animal decreased after approx. 3 months after the 2^nd^ operation. We were able to find an abscess in the Tympanon region but we could not correlate the appearance of the abscess in the Tympanon with the treatment.

Four animals deceased suffering from infections of the intestine and the respiratory system, thromboembolic complications and a myocardial infraction. Similar adverse events are reported in literature. Gotterbarm et al. [14] reported that one GM had to be taken out of the study 26 weeks after surgery due to a minor stroke. Several days (161 days) after surgery, [58] reported one animal that died of a respiratory infection. Birck et al. [59] reported the death of two GM due to a thromboembolic complication within 2 months after Roux-en-Y gastric bypass surgery; the animals were treated with central venous catheters. Murray and Fleming [58] lost 2 animals in their study 62 animals that included, one when anesthesia was induced in a study for ACL reconstruction in pigs. In a former study with 12 pigs they excluded 2 animals from the study [74]) though the complications were probably induced by the treatment. Wang et al. [43] lost 1 of 12 young pigs, also due to anesthesia in addition to 3 animals suffered postoperatively from wound infections which amounted to 33% of the pigs included in the study.

Gotterbarm et al. [75] reported 1 severe infection in 22 GM (4.6%) and [76] reported about 1 severe infection in 8 GM (12.5%) leading to the premature killing of the affected animal. Schwarz et al. [77] also reported one animal lost in a study dealing with the osseointegration of titanium implants in 20 GM (5%).

In the presented study we have a rate of 9% (7/77) where severe complications caused the loss of the animals; we included the 10 animals of the NAT group in this statistics as we saw that deaths in the treated groups were caused by diseases independently from the surgical treatment. In addition we were not able to include some GM in the study one due to a histologically proven breast carcinoma and another one due to spontaneous death. The events occurred during the time of acclimatization of the animals after shipping.

### Drop outs

In 3 animals only one knee was able to be treated during the 2^nd^ operation due to a subcutaneous purulent abscess which we noticed before opening the knee joint. These knee joints and the related (spontaneous) regenerative tissue samples were noted as “drop outs” but nonetheless included in the list of samples and blinded to get the complete number of samples. However, the samples had to be excluded from the statistics. Thus, the sample size is reduced in special cases but the exclusion of the animal as a whole was not acceptable to us due to reasons of animal welfare because the samples of the operated side were still available.

### Infections

Local infections after surgery in the area of the wound are explainable as the distance to the ground is short and the animals were housed in straw for their welfare [10]. As the wound is near the belly of the animal it can also be affected leading to a secondary infection.

We think that the closure of the wound layer by layer may help to avoid contamination of the joints (30).

However, we found a higher rate of signs of infections after the 2^nd^ operation than after the 1^st^ one reflecting the longer duration of the surgery and also the higher stress on the tissue due to more manipulations. Most infections were superficial in the region of the wounds mainly occurring on the scar and in the stitch channels. Thus in most cases it was possible to treat the infections locally.

In some cases, further treatment of abscess- like formations was not necessary as they healed spontaneously, including one on the facial region of an animal. Abscess formations in the facial region seem to arise more often, as [58] treated an abscess near the jaw of a Yucatan–minipig and [77] held an abscess in the facial region accountable for an infection of one implantation site.

In the present study the administration of antibiotics was necessary in a few cases and a surgical treatment had to be performed in one case, a situation similar to the one reported by [18].

The seroma we found when opening the joints for the 2^nd^ operation did not bother the animals in an obvious way. Only in a few cases we found germs. They did not seriously jeopardize the surgery as we did not find empyema in the knee joints later on. Through the surgical approach to the knee joint in the 2^nd^ operation the seroma seemed to have been drained successfully.

### Histologic outcome of the treatments and controls

The results achieved by the scoring system according to O´Driscoll et al. [39] represent the effect of the treatments as well as the validity of the model. However we detected a wide spread of the values indicating that some individuals may have a higher potential for regeneration then others. The outlier in the NAT group represents a consequence of blinded examination, even though if the final scoring values were harmonized in a consensus meeting (Fig 7).

In this report we want to focus more on the usefulness of the study design than on the results in terms of the outcomes of used implants. However, one can see that the outcomes of the groups without autologous cells (M24w and M48w) were closer to the condition of the natural cartilage of the NAT group than the groups that received matrices with cells. The latter showed significant differences after ½ and 1 year (Fig 7). Schneider et al. [33] compared the outcome of Col I scaffolds laden with or without autologous cells with spontaneous regenerated cartilage tissue in a comparable animal model and implants after an observation time up to 1 year. The authors reported that the implants had similar values according to the O’Driscoll score but better scores than the regenerated tissue in the defects with a diameter of 6.3 mm, which were left empty.

Thus the question of the differentiability of the results becomes more interesting. In the present study this is reflected by the clear differences between the means of the treated groups and the NAT group (Fig 7) also when the differences are not significant for the M24w and the M48w groups. But a differentiation between the groups with cells (MC24w and MC48w) and the groups without cells (M24w and M48w) is possible when they are compared with the NAT group. This is not the case when they are compared with a group where the defects were left empty after 24 weeks (E24w group).

Obviously, the GM does not have the potential to regenerate a 6 mm defect spontaneously, even after nearly one year (Fig 7). Therefore, the model could also be used with the empty defects as controls as Schneider et al. [33] did. But the results show that the probability of detecting differences to the treated groups increases after an observation time of nearly 1 year and not after ½ year. This renders the model unattractive in terms of resource consumption, but this drawback could be overcome by including a NAT group as shown in the present study. Thus, the results accentuate the usefulness of the implementation of a NAT group as control.

### Degenerative changes in the articular cartilage close to the defects and in the knee joints

Looking at the ratings we were able to determine the impact of the surgical procedures on the articular cartilage of the trochleae adjacent to the area of operation (Fig 10), the cartilage of the condyles far from the area of operation (Fig 11) and to the complete joint (Fig 8 and Fig 9). Looking at the NAT group we noticed, that the stifle joints did not seem to be free from degenerative changes of the articular cartilage. The cartilage of the condyles showed significantly more degenerative changes than that of the trochleae in the NAT group. It was surprising to see that the condyles of the NAT group showed a tendency for a higher degree of degenerative changes than that of treated groups (Fig 11). The group treated with matrices alone revealed a significantly better condition of the condyles after ½ year but after 1 year it was the group with cell laden matrix that revealed a better condition (Fig 11). It is speculation if a healing effect could have occurred. But we think that the results confirm the assumption that the trochlea is more robust against the occurrence of degenerative changes, even after two surgical treatments especially, as the tibio–femoral joint was not touched. Thus, we see further evidence that the area of the trochlea appears to be the better place for a cartilage defect model than the condyles (Fig 11).

One has to keep in mind that skeletally adult GM may already have degenerative changes in the knee joints when starting a study but the maturity makes this animal model more comparable with the human situation (Chaganti and Lane 2011 [67], Prieto-Alhambra et al. [68]).

But age, weight and weight gain did not seem to have seriously influenced the development of degenerative changes of the articular cartilage. We found two conspicuous data in the age in the MC24w group and in the starting weight in the E24w group. However, negative correlations were detected whereas the opposite would have been likely.

According to the results of the radiographs we cannot either assume that the presented surgical performance had seriously altered the entire stifle joints (Fig 8).

However, macroscopically the M48w group was significantly worse than the NAT group in terms of degenerative changes even though the worst reactions were macroscopically seen in the E48w group (Fig 9).

### Hypertrophic reactions (HT)

As described in literature we found HT after treatment [1], [75], [78], [79]. However it seems that the development of hypertrophic reactions is not limited to cell laded matrices as it could even appear when the cartilage defects were left empty and untreated. As we identified less HT after 1 year than after ½ year, we assume that the HT developed within the first time period after surgery.

A drawback regarding this issue is the fact, that we were not able to analyze the hypertrophic tissue as we had no tool to exactly measure the extension of hypertrophy in accordance with the clinically used MRI based determination [79] or arthroscopic intervention [80]. However, the presented animal model seems to be suitable for addressing this issue of regenerative treatments of articular cartilage.

### Statistics

The outlined procedure for randomization of the samples in the presented study seems a little complicated. But it was helpful to overcome the disadvantage that there were 8 defect locations but 10 animals, which were needed as sample size (Fig 1). Thus, it was possible to perform a random allocation of each sample even when two defect locations existed twice; randomization and blinding of the samples were necessary following the by the ARRIVE guidelines [21].

It was a challenge to create the appropriate statistical analysis plan in concordance with the aim of the planned study, more so as there are various analyses available and we had to find out which procedure of analysis could be the most appropriate.

We intended to develop a procedure which would be helpful for the calculation sample size and focus on the evaluation afterwards. Fig 5 confirms our sample size calculation as it shows that an increase in the sample size does not enhance the power significantly. Fig 5 shows the impact of a higher ES.

Using ESs enables researchers to find the most meaningful results amongst treatments and analyses.

Cohen’s “d” is a family of statistics for standardized differences between observations. The variations of Cohen’s “d” consist of differences between independent or dependent observations, whether the variances are assumed to be known or are estimated, and, in the case of independence, whether the variances are pooled or not [25]. Further variations such as corrections for bias etc. are also described in the literature [81]. Globally Cohen’s “d” can be simply defined as the difference in the observations divided by the standard deviation of the difference in the observations.

It may be noted that Cohen’s “d” is equivalent to the standardized normal deviate also known as the z-score of a standard normal distribution. In terms of the testing of a hypothesis, an interpretation of Cohen’s “d” is “The degree to which H_0_ is false is indexed by the discrepancy between H_0_ and H_1_ and is called the ES” [26]. Each statistical calculation has its own ES. Since the ES is a scale free continuous index it can be used to compare parameters within or across studies.

We performed exemplarily a comparison of the detected p–values and the ES in terms of a ranking in classes (e. g. < 0.5, 0.5–0.8 and > 0.8) with the results of the histologic evaluation (Table 2). Looking at the results of the E24w and the E48w groups the difference was not significant (p = 0.0545) but looking at the ES it can be ranked in the higher (high) class of ES. On the contrary, the difference between the MC24w and the NAT group was significant (p = 0.0106) but can be ranked only in a medium class of the ES (Table 2). The presented estimations are quite sound as in all addressed groups (E24w, E48w, MC24w and the NAT group) the complete data set of 10 samples was available. Thus one can see that the description of the ES can enhance the assessability of the values and the classification of the results. It can subsequently help to determine the quality with regard to the assessed treatment or type of analysis.

But it might also be helpful to consider a number of ESs to determine a sample size for an acceptable level of power in the planning stage of an experiment, especially in a case where different measurements are taken under varying conditions, with little knowledge of the true variability of these measurements. Fig 5 shows that the ES is more relevant than the sample size as a small ES has nearly no effect on the power whereas a higher ES will enhance the power also with a higher number of samples.

However, we think that the most notable advantage of the ESs is that the ESs can be ranked and compared with ESs calculated in other experiments and studies (Table 2).

### The 3 Rs

At the beginning of the study we performed several examinations and developed processes using cadaver specimens from cadavers at first from the slaughter house and then we used dead GM. Thus “insentient” material was used which can be interpreted as *Replacement* according to [23, 24]. Finally, pilot animals were used, which can be seen as a process of *Refinement* regarding the animals included in the study [23, 24]. Thus, the final protocol of the study was developed using specimens from cadavers [30] and ultimately using of animals as pilots. This resulted in a refinement of the treatment.

The demands and execution of the *Reduction* principle [23] is defined in a more sophisticated manner, as reduction is not strictly the minimization of animal numbers used instead “enough animals have been used” [23, 24].

The animal model in the presented study can help to fulfill the demand for reduction also in terms of the numbers of animals which had to be included due to the arrangement of the surgical treatment and the determination of the groups (Table 1). One should take into consideration that the number of techniques that can be used to characterize the properties of natural and regenerated cartilage is growing rather than decreasing [9]. Thus more samples and finally more animals are needed, as traditional types of analyses are not replaced by new ones at this point in time [9]. The presented model provides 8 samples all resembling each other, originating from one animal and possible differences caused by different locations in the trochlea can be compensated by a randomization procedure as described above. One conspicuous detail in the presented model is the inclusion of the NAT group consisting of untreated animals. It is not yet proven, if such a group is always needed. But the effect of the treatment or the surgical approach can be identified by doing that as the comparison between “inner controls” from operated knee joints can also be performed with the tissue of untreated knees as “outer controls”. The most important reason for including the NAT group was to get samples of articular cartilage that are completely pristine and are the best suited as control tissue. Another point is that the welfare of the animals of a NAT group which included 14% of the animals in the presented study is not compromised by any distress resulting from surgical treatment other than the sampling of blood under sedation. However, at a first glance the costs rise when a NAT group is included but then the comparison with complete uncompromised and natural cartilage is possible.

At a second glance one can see, that the presented type of animal model is cost-effective, too, as animals can be saved:

If in a study one regenerate of articular cartilage from one joint was compared to a natural sample of cartilage from the contralateral joint 80 animals would have to be included for one group when the sample size calculation delivered the number of 10 for one of 8 types of analyses. Thus, the number of animals, which had to be included, would rise to 480 animals with 6 groups and 2 healing periods. With the presented model we reduced the number of animals by 85%. The possibility to reduce the number of animals by using of a model which delivers several samples from one animal is evident, as more defects can be created in one animal. However, we think that 4 defects with a diameter of 6 mm is the upper limit of defects that can be placed in one trochlea of GM because of concerns regarding the surgical approach, the biomechanical stability or the later development of severe degenerative changes of the joint. An alternative model would provide 4 defects from one knee and 4 controls from the (untreated) contralateral knee joint. This would require 120 animals that would have to be operated if 8 samples had to be created for 8 different types of analyses with a sample size of 10 in each of 6 groups. That number of animals needed would be 1.7 times higher, or in other words 71% more animals than we needed for the presented study (n = 70). This benefit would still exist, if a further NAT group for both time points (here 24 weeks and 48 weeks) had been included bringing the number up to 80 animals in the presented model. However, we think that the decision to include only one NAT group was sound even, if it was housed for just 24 weeks in order to keep the numbers of animals as low as possible while still observing the expected “precision” [23, 24].

The postoperative observations confirm that the animals were not distressed more than they were in comparable studies when 8 defects per animal were set.

Thus, we think that we were able to follow the principles of [23, 24] with the presented animal model even though fewer animals were involved [22] without an evident increase in distress in the operated animals but with high “efficiency” by “generating maximum scientific or medical results from expenditures of monetary and animal resources, facilities, and personnel” [23, 24].

## Conclusion

Summarizing the data collected in the presented study we believe that the application of 8 partial thickness defects allocated to both knee joints of one Göttingen Minipig in a symmetric manner makes the presented large animal model useful for producing samples of regenerated articular cartilage in a high number. It opens the door to complex analyses also due to the fact that the double number of controls can be provided. From the scientific and economic point of view the model becomes efficient because of the inclusion of a group of untreated animals (NAT). It could help to histologically differentiate the outcomes of treatments in a shorter observation time than it is possible in comparison with spontaneously regenerated articular cartilage tissue. The cartilage of the trochlea proved to be robust against degenerative changes despite the two surgeries and despite the existence of a prior primary degeneration. The use of the effect size is a statistical parameter that can help make the values of several types of treatment and analyses comparable. We think that the presented model has the potential to improve further standardization of research while complying with the regulatory requirements for regenerative therapies of articular cartilage regarding the welfare of the animals, as it is cost effective and in accordance with 3R requirements.

## Supporting information

S1 Checklist ARRIVE Guidelines In the column “Section/Paragraph” the subheadings and / or the paragraphs are listed where the items are addressed in the text body of the paper. (PDF)

S1 Table Raw data The descriptions of the groups are given in the paper (Table 1). The table provides the data of the age of the animals, their weight when the trial started and their weight gain during the observation periods. The values are given for the macroscopic scoring of the knee joints, the histological scoring of the regenerates (according to O’Driscoll et al.), the cartilage of the condyles and the trochlea adjacent to the defects together with the radiologic condition of the joints according to Kellgren and Lawrence. The durations of the first and the second operations are given in minutes also for the seven lost animals.
(XLSX)
